# New Cyclotetrapeptides and a New Diketopiperzine Derivative from the Marine Sponge-Associated Fungus *Neosartorya glabra* KUFA 0702

**DOI:** 10.3390/md14070136

**Published:** 2016-07-20

**Authors:** War War May Zin, Suradet Buttachon, Tida Dethoup, Carla Fernandes, Sara Cravo, Madalena M. M. Pinto, Luís Gales, José A. Pereira, Artur M. S. Silva, Nazim Sekeroglu, Anake Kijjoa

**Affiliations:** 1 *ICBAS*—Instituto de Ciências Biomédicas Abel Salazar, Universidade do Porto, Rua de Jorge Viterbo Ferreira, 228, 4050-313 Porto, Portugal; wwmzin.chem.yu@gmail.com (W.W.M.Z.); nokrari_209@hotmail.com (S.B.); lgales@ibmc.up.pt (L.G.); jpereira@icbas.up.pt (J.A.P.); 2 Interdisciplinary Centre of Marine and Environmental Research (CIIMAR), Rua dos Bragas 289, 4050-313 Porto, Portugal; cfernandes@ff.up.pt (C.F.); scravo@ff.up.pt (S.C.); madalena@ff.up.pt (M.M.M.P.); 3 Department of Plant Pathology, Faculty of Agriculture, Kasetsart University, 10240 Bangkok, Thailand; agrtdd@ku.ac.th; 4 Laboratório de Química Orgânica, Departamento de Ciências Químicas, Faculdade de Farmácia, Universidade do Porto, Rua de Jorge Viterbo Ferreira, 228, 4050-313 Porto, Portugal; 5 Instituto de Biologia Molecular e Celular (IBMC), Universidade do Porto, Rua de Jorge Viterbo Ferreira, 228, 4050-313 Porto, Portugal; 6 Departamento de Química & QOPNA, Universidade de Aveiro, 3810-193 Aveiro, Portugal; artur.silva@ua.pt; 7 Medicinal and Aromatic Plant Programme, Plant and Animal Sciences Department, Vocational School, Kilis Aralık University, 79000 Kilis, Turkey; nsekeroglu@gmail.com

**Keywords:** *Neosartorya glabra*, marine-derived fungus, *Mycale* sp., cyclotetrapeptides, sartoryglabramides A and B, diketopiperazines, fellutanine A epoxide

## Abstract

Two new cyclotetrapeptides, sartoryglabramides A (**5**) and B (**6**), and a new analog of fellutanine A (**8**) were isolated, together with six known compounds including ergosta-4, 6, 8 (14), 22-tetraen-3-one, ergosterol 5, 8-endoperoxide, helvolic acid, aszonalenin (**1**), (3*R*)-3-(1*H*-indol-3-ylmethyl)-3,4-dihydro-1*H*-1,4-benzodiazepine-2,5-dione (**2**), takakiamide (**3**), (11a*R*)-2,3-dihydro-1*H*-pyrrolo[2,1-*c*][1,4]benzodiazepine-5,11(10*H*,11a*H*)-dione (**4**), and fellutanine A (**7**), from the ethyl acetate extract of the culture of the marine sponge-associated fungus *Neosartorya glabra* KUFA 0702. The structures of the new compounds were established based on extensive 1D and 2D spectral analysis. X-ray analysis was also used to confirm the relative configuration of the amino acid constituents of sartoryglabramide A (**5**), and the absolute stereochemistry of the amino acid constituents of sartoryglabramide A (**5**) and sartoryglabramides B (**6**) was determined by chiral HPLC analysis of their hydrolysates by co-injection with the d- and l- amino acids standards. Compounds **1**–**8** were tested for their antibacterial activity against Gram-positive (*Escherichia coli* ATCC 25922) and Gram-negative (*Staphyllococus aureus* ATCC 25923) bacteria, as well as for their antifungal activity against filamentous (*Aspergillus fumigatus* ATCC 46645), dermatophyte (*Trichophyton rubrum* ATCC FF5) and yeast (*Candida albicans* ATCC 10231). None of the tested compounds exhibited either antibacterial (MIC > 256 μg/mL) or antifungal activities (MIC > 512 μg/mL).

## 1. Introduction

**Figure 1:**
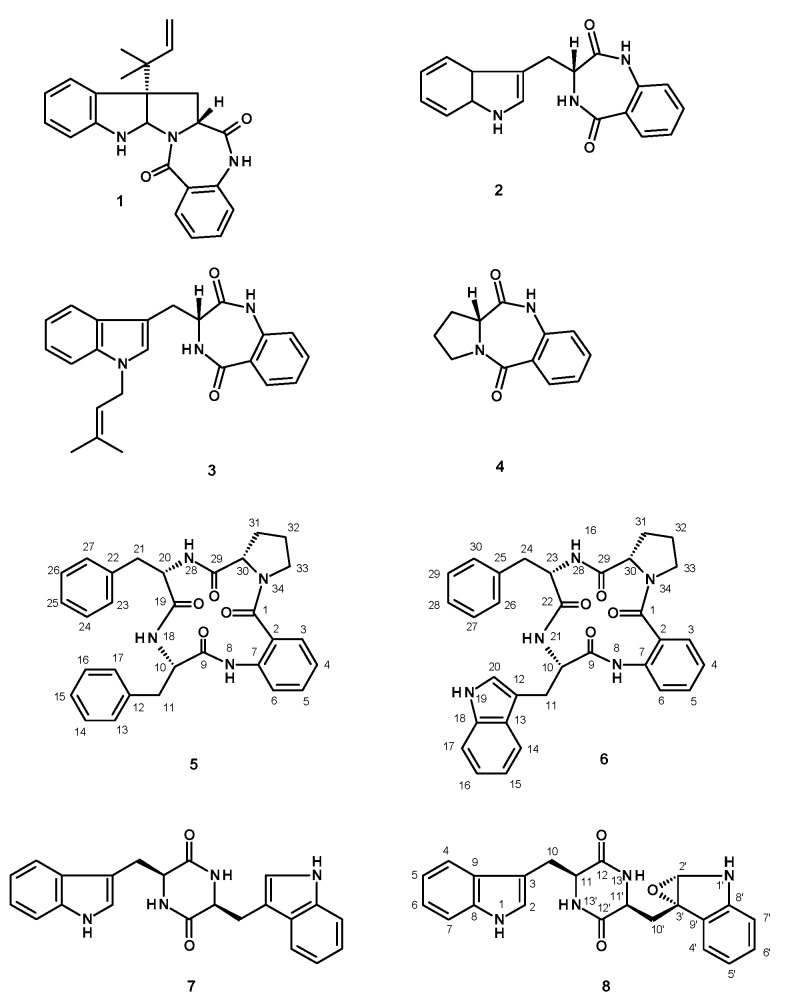
Secondary metabolites isolated from the ethyl acetate extract of the culture of *N. glabra* KUFA 0702.

Although the chemical constituents of the fungi of the genus *Neosartorya*, a teleomorphic state of *Aspergillus* section *Fumigatus*, have not previously been intensively investigated [1], there are currently three reports on the secondary metabolites and their biological activities of *Neosartorya glabra* (Fennell & Raper) Kozakiewicz. Jayasuriya et al. first described isolation of three new antibacterial bicyclic lactones, glabramycins A–C, from *N. glabra* isolated from a soil sample collected from Candamia, Spain, by antisense screening [2]. However, it is only very recently that the synthesis and revision of the relative configuration of glabramycin B were achieved [3]. Kijjoa et al. described isolation of three new reverse prenylated indole derivatives, sartoryglabrins A–C, and their in vitro growth inhibitory activity against three human cancer cell lines, from the Thai collection of a soil-derived *N. glabra* [4]. Recently, Liu et al. reported isolation of two new polyketides, neosarphenols A and B, together with six known polyketides and two known meroterpenoids, from the crude ethyl acetate extract of *N. glabra* CGMCC 32286 [5]. During our ongoing search for bioactive secondary metabolites from members of the genus *Neosartorya* and our pursuit for natural antibiotics from marine-derived fungi, we have investigated the secondary metabolites of a Thai collection of *N. glabra* KUFA 0702, isolated from the marine sponge *Mycale* sp., collected from the coral reef at Samaesarn Island in the Gulf of Thailand. The ethyl acetate extract of its culture furnished three new compounds including two new cyclotetrapeptides, sartoryglabramides A (**5**) and B (**6**), and a new analog of fellutanine A (**8**), in addition to the previously reported ergosta-4,6,8 (14), 22-tetraen-3-one [6], ergosterol 5, 8-endoperoxide [7], helvolic acid [8], aszonalenin (**1**) [9], (3*R*)-3-(1*H*-indol-3-ylmethyl)-3, 4-dihydro-1*H*-1,4-benzodiazepine-2,5-dione (**2**) [10], takakiamide (**3**) [11], (11a*R*)-2,3-dihydro-1*H*-pyrrolo[2,1-*c*][1,4]benzodiazepine-5,11(10*H*,11a*H*)-dione (**4**) [12], and fellutanine A (**7**) [13,14] (Figure 1). Compounds **1**–**8** were tested for their antibacterial activity against Gram-positive (*Escherichia coli* ATCC 25922) and Gram-negative (*Staphyllococus aureus* ATCC 25923) bacteria, as well as for their antifungal activity against filamentous (*Aspergillus fumigatus* ATCC 46645), dermatophyte (*Trichophyton rubrum* ATCC FF5) and yeast (*Candida albicans* ATCC 10231).

## 2. Results and Discussion

**Figure 2:**
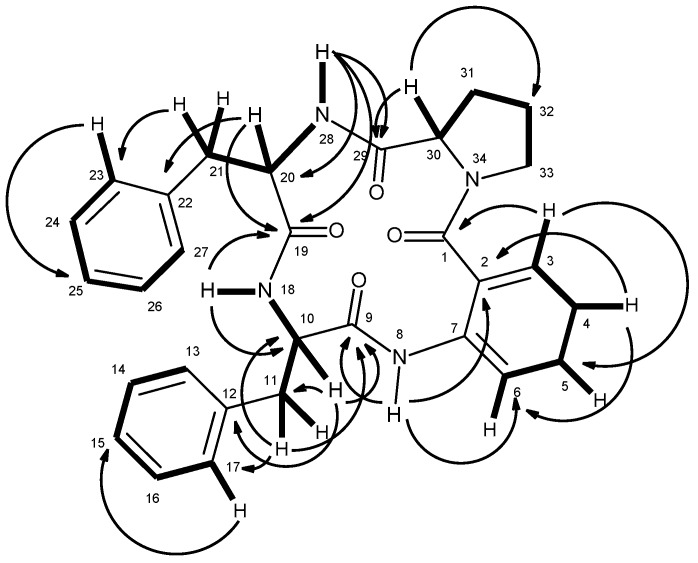
Key COSY (—) and HMBC (→) correlations of compound **5**.

Compound **5** was isolated as white crystals (mp, 146–148 °C), and its molecular formula C_30_H_30_N_4_O_4_ was established on the basis of the (+)-HRESIMS *m*/*z* 511.2365 [M + H]^+^, indicating eighteen degrees of unsaturation. The IR spectrum showed absorption bands for amine (3447 cm^−1^), amide carbonyl (1655 cm^−1^) and aromatic (1622, 1587, and 1526 cm^−1^). The ^13^C NMR (Supplementary Materials, Figure S10), DEPTs and HSQC spectra (Table 1, Supplementary Materials, 12) revealed the presence of four amide carbonyls (δ_C_ 170.2, 169.9, 168.8, 166.5), four quaternary sp^2^ (δ_C_ 138.3, 137.3, 136.5, 124.8), fourteen methine sp^2^ [δ_C_ 130.4, 129.6 (2C), 129.1 (2C), 128.1 (2C), 128.0 (2C), 126.6, 126.3, 126.0, 122.4, 120.4], three methine sp^3^ (δ_C_ 62.2, 55.2, 54.4), and five methylene sp^3^ (δ_C_ 49.4, 37.1, 34.7, 28.3, 24.6). The ^1^H NMR spectrum (Table 1, Supplementary Materials, Figure S9) revealed three NH signals at δ_H_ 9.40, s, 8.49, d (*J* = 7.8 Hz) and 7.41, d (*J* = 9.8 Hz), the signals of four aromatic protons of anthranilic acid at δ_H_ 8.31, dd (*J* = 7.9, 0.5 Hz, H-6), 7.55, dd (*J* = 7.7, 1.3 Hz, H-3), 7.48, ddd (*J* = 7.9, 7.9, 1.4 Hz, H-5) and 7.16, dd (*J* = 7.9, 7.7 Hz, H-4) [15]. The anthranilic acid residue was linked to the phenylalanine residue, through the amino group of the former and the carboxyl group of the latter, since the HMBC spectrum (Supplementary Materials, Figure S12) showed correlations of the NH signal at δ_H_ 9.40, s (NH-8) to the carbonyl carbon at δ_C_ 168.8 (C-9), C-2 (δ_C_ 124.8), C-6 (δ_C_ 120.4), of the methine proton at δ_H_ 4.36, ddd (*J* = 8.4, 7.8, 5.3 Hz, H-10) to C-9, C-11 (δ_C_ 34.7), C-12 (δ_C_ 138.3), of the methylene protons at δ_H_ 2.97, dd (*J* = 13.9, 8.4 Hz, H-11a) and 3.23, dd (*J* = 13.9, 5.3 Hz, H-11b) to C-9, C-10 (δ_C_ 55.2), C-12, C-13/C-17 (δ_C_ 129.6). The COSY spectrum also showed correlation (Supplementary Materials, Figure S11) of H-10 to H_2_-11 of this phenylalanine residue (Table 1 and Figure 2). That this phenylalanine residue (Phe-I) was linked to another phenylalanine residue (Phe-II) was corroborated by the COSY correlation of H-10 to the proton doublet at δ_H_ 8.49, d (*J* = 7.8 Hz, NH-18), as well as by the HMBC correlations of NH-18 to C-10 and the carbonyl carbon at δ_C_ 169.9 (C-19), of the methine proton signal at δ_H_ 4.58, ddd (*J* = 9.8, 8.9, 7.3 Hz, H-20) to C-19, C-21 (δ_C_ 37.1), C-22 (δ_C_ 137.3), of the methylene proton signals at δ_H_ 2.71, dd (*J* = 13.5, 8.9 Hz, H-21a)/2.94 dd (*J* = 13.5, 7.3 Hz, H-21b) to C-22, C-19, C-20 (δ_C_ 54.4), and C-23/C-27 (δ_C_129.1) (Table 1 and Figure 2). This was further supported by the COSY correlations of H-20 to H_2_-21 and the proton doublet at δ_H_ 7.41 (*J* = 9.8 Hz, NH-28). The existence of the proline residue was evidenced not only by the COSY correlations of the double doublet at δ_H_ 4.20 (*J* = 9.8, 2.3 Hz, H-30; δ_C_ 62.2) to the multiplets at δ_H_ 1.54 and 2.12 (H_2_-31, δ_C_ 28.3), of the multiplet at δ_H_ 1.89 (H_2_-32; δ_C_ 24.6) to H_2_-31 and the double doublet at δ_H_ 3.70 (*J* = 17.6, 9.6 Hz, H-33a; δ_C_ 49.4) and a multiplet at δ_H_ 3.63 (H-33b; δ_C_ 49.4) but also by the HMBC correlations of H-30 to the carbon signals at δ_C_ 170.2 (CO-29), δ_C_ 28.3 (C-31) and δ_C_ 24.6 (C-32), and of H-33a to C-30 (δ_C_ 62.2), C-32, of H_2_-31 to C-29 and C-30, respectively (Table 1 and Figure 2). That the proline residue was connected to the Phe-II residue, through the carbonyl of the former and the amino group of the latter, was corroborated by the HMBC correlation of NH-28 to CO-29. Since there are only three NH signals, the nitrogen of the pyrrolidine ring of the proline residue was linked to the carbonyl group (C-1) of anthranilic acid. This was corroborated by the HMBC correlations of H-3 to CO-1 (δ_C_ 166.5), and of NH-8 to C-2 (δ_C_ 124.8) and C-6 (δ_C_ 120.4) (Table 1 and Figure 2). Therefore, combining this information, it was possible to conclude that **5** was cyclo (anthranilic acid-Phe-Phe-Pro).

**Figure 3:**
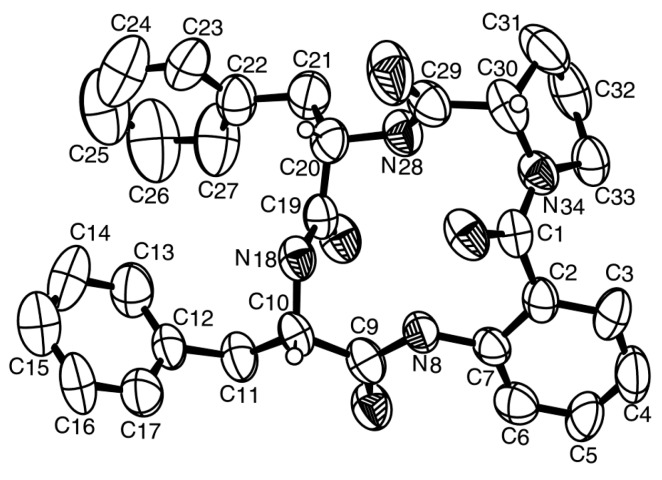
Ortep view of compound **5**.

Since **5** was obtained as a suitable crystal for X-ray diffraction, the stereochemistry of its amino acid residues was tentatively determined by X-ray analysis, and the ORTEP view shown in Figure 3 revealed that Phe-I, Phe-II and Pro have the same relative configuration. However, since the flack *x* parameter (0.3) did not guarantee the absolute confidence of the absolute configurations, the stereochemistry of the amino acid residues of **5** was confirmed by a chiral HPLC analysis of its acidic hydrolysate, using appropriate d- and l-amino acid standards, according to the previously described method [15]. The enantioseparations of the standard amino acids were successfully performed with the Chirobiotic T column under reversed-phase elution conditions [16]. The elution order of the enantiomers of all the standards amino acids was confirmed by injecting the solutions of the enantiomeric mixtures and then each enantiomer separately at a flow rate of 1 mL/min (Supplementary Materials, Table S1). As predicted, the d-enantiomer was always more strongly retained than the corresponding l-enantiomer on Chirobiotic column [16]. The retention times (t_R_ min) for standards amino acids, using MeOH: H_2_O (80:20 *v*/*v*) as mobile phase, at a flow rate of 1.0 mL/min, and with UV detection set at 210 nm, were l-Phe (3.8) and d-Phe (5.0), l-Pro (6.7) and d-Pro (20.1). Based on mix HPLC analyses of the acidic hydrolysate with standard d- and l-amino acids (co-injection) (Supplementary Materials, Figure S27 and Table S1), compound **5** was elucidated as cyclo (anthranilic acid-l-Phe-l-Phe-l-Pro). Since compound **5** is a new compound, we have named it sartoryglabamide A.

**Figure 4:**
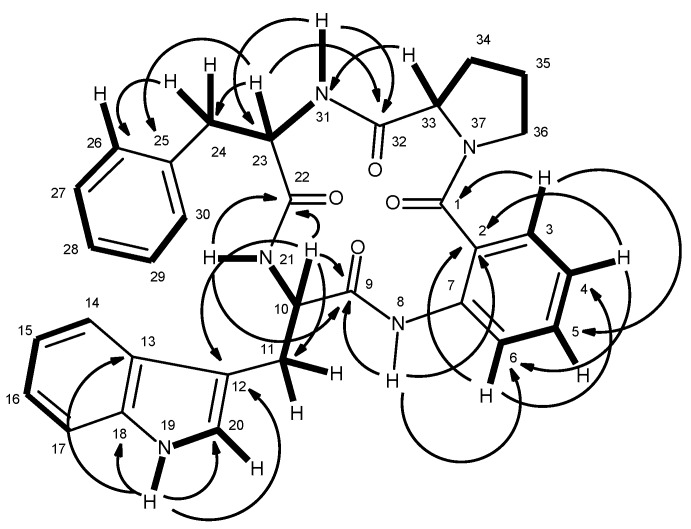
Key COSY (—) and HMBC (→) correlations of compound **6**.

Compound **6**, which was also isolated as white solid (mp, 190–192 °C), exhibited the [M + H]^+^ peak at *m*/*z* 550.2501 [(+)-HRESIMS], corresponding to C_32_H_32_N_5_O_4_ (calcd. 550.2454). Therefore, the molecular formula C_32_H_31_N_5_O_4_ was attributed to compound **6**, which indicated twenty degrees of unsaturation. Like compound **5**, the IR spectrum of **6** showed absorption bands for amine (3417 cm^−1^), amide carbonyl (1649 cm^−1^) and aromatic (3058, 1620, 1588, 1526 cm^−1^). With some exceptions, the general features of the ^1^H and ^13^C spectra of compound **6** resembled those of **5**. The ^13^C NMR (Supplementary Materials, Figure S15), DEPTs and HSQC spectra (Table 2, Supplementary Materials, Figure S17) displayed signals of four carbonyls (δ_C_ 170.2, 170.1, 169.0, 166.4), six quaternary sp^2^ (δ_C_ 137.4, 136.3, 136.0, 127.7, 125.2, 110.2), fourteen methine sp^2^ [δ_C_ 130.4, 129.0 (2C), 128.1 (2C), 126.5, 126.3, 124.0, 122.6, 120.8, 120.7, 118.5, 118.2, and 111.3], three methine sp^3^ (δ_C_ 62.1, 54.6, 54.3), and five methylene sp^3^ (δ_C_ 49.4, 37.0, 28.3, 24.9, 24.6). Unlike compound **5**, the ^1^H NMR spectrum of **6** (Table 2, Supplementary Materials, Figure S15), exhibited four NH signals at δ_H_ 10.82, d (*J* = 1.8 Hz), 9.25, s, 8.42, d (*J* = 7.9 Hz) and 7.38, d (*J* = 10.0 Hz). Similar to compound **5**, the presence of the proline residue was corroborated by the presence of the coupling system of the protons from H-33 to H_2_-36 [(δ_H_ 4.15, dd, *J* = 9.0, 2.0 Hz, H-33; δ_C_ 62.1), δ_H_ 1.45 m and 2.09, m (H_2_-34; δ_C_ 28.3), δ_H_ 1.86 m (H_2_-35; δ_C_ 24.6), and δ_H_ 3.55 m and 3.67, m (H_2_-36; δ_C_ 49.4)] as well as by the HMBC correlation of H-33 to the carbonyl carbon at δ_C_ 170.2 (C-32), while the presence of the phenylalanine residue was supported by the coupling system from H_2_-24 (δ_H_ 2.66, dd, *J* = 13.6, 10.0 Hz, and 2.92, dd, *J* = 13.6, 6.4 Hz; δ_C_ 37.0) through H-23 (δ_H_ 4.61, ddd, *J* = 10.0, 10.0, 6.4 Hz; δ_C_ 54.6) to NH-31 (δ_H_ 7.38, d, *J* = 10.0 Hz), as observed in the COSY spectrum, as well as by the HMBC correlations from H-23 to C-24 (δ_C_ 37.0) and C-25 (δ_C_ 137.4), of H_2_-24 to C-23 (δ_C_ 54.6), C-25, C-26/30 (δ_C_ 129.0)(Table 2 and Figure 4). Like compound **5**, the HMBC correlation of the amine proton at δ_H_ 7.38, d (*J* = 10 Hz, NH-31) to C-32 confirmed the linkage of the carbonyl group of the proline residue (C-32) to the amino group of the phenylalanine residue (N-31). Similarly, the nitrogen of the pyrrolidine ring of the proline residue (N-37) was linked to the carbonyl group of anthranilic acid (C-1, δ_C_ 166.4). That one of the phenyl residues of **5** was replaced by a tryptophan residue in **6** was substantiated by the presence of the indole system, which was characterized by the coupling system of H-14 (δ_H_ 7.58, d, *J* = 7.9 Hz, δ_C_ 118.5) through H-17 (δ_H_ 7.34, d, *J* = 8.0 Hz, δ_C_ 111.3), as observed in the COSY spectrum (Table 2 and Figure 4, Supplementary Materials, Figure S16), and also by the HMBC correlations from NH-19 (δ_H_ 10.82, brs) to C-12 (δ_C_ 110.2), C-13 (δ_C_ 127.7), C-18 (δ_C_ 136.0) and C-20 (δ_C_ 124.0) (Table 2 and Figure 4, Supplementary Materials, Figure S18), as well as of the ethylamino moiety, as evidenced by the coupling system from H_2_-11 (δ_H_ 3.14, dd, *J* = 14.7, 6.7 Hz and 3.32 dd, *J* = 14.7, 5.9 Hz; δ_C_ 24.9) through H-10 (δ_H_ 4.52, ddd, *J* = 7.9, 6.7, 5.9 Hz; δ_C_ 54.3) to NH-21 (8.42, d, *J* = 7.9 Hz) (Table 2 and Figure 4). That the tryptophan residue was linked to the phenylalanine residue, through the amino group of the former and the carbonyl group of the latter, was corroborated by the HMBC correlations of NH-21 to the carbonyl carbons at δ_C_ 170.1 (C-22) and 169.0 (C-9), as well as of H-10 to C-9, C-11, C-12 and C-22. Finally, the amino group of the anthranilic acid residue was linked to the carbonyl group of the tryptophan residue was supported by the HMBC correlations of NH-8 (δ_H_ 9.25, s) to C-2 (δ_C_ 125.2), C-6 (δ_C_ 120.7) and C-9. Therefore, **6** was identified as cyclo (anthranilic acid-Trp-Phe-Pro).

The absolute stereochemistry of the amino acid residues of compound **6** was also determined by chiral HPLC analysis of its acidic hydrolysate, using appropriate d- and l-amino acids standards. The retention times (t_R_ min) for standards amino acids, using MeOH: H_2_O (80:20 *v*/*v*) as mobile phase, at a flow rate of 1.0 mL/min, and with UV detection set at 210 nm, were l-Phe (3.8) and d-Phe (5.0), l-Pro (6.7) and d-Pro (20.1), l-Trp (4.5) and d-Trp (5.2). Based on mix HPLC analyses of the acidic hydrolysate with standard d- and l-amino acids (co-injection) (Supplementary Materials, Figure S28, Table S1), compound **6** was elucidated as cyclo (anthranilic acid-l-Trp-l-Phe-l-Pro). Since compound **6** is also a new compound, we have named it sartoryglabamide B.

**Figure 5:**
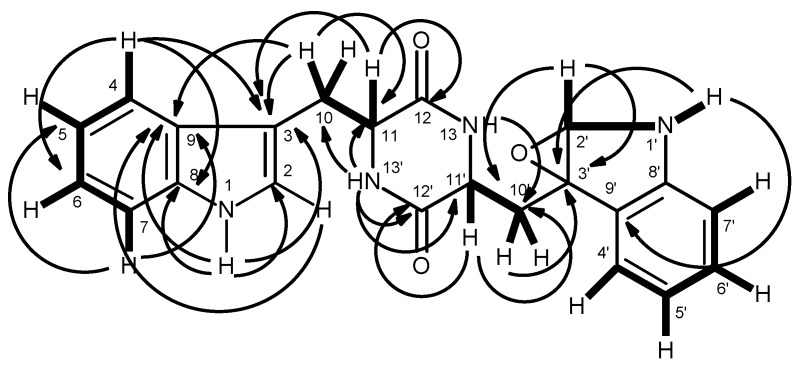
Key COSY (—) and HMBC (→) correlations of compound **8**.

Compound **8** was isolated as pale yellow viscous mass, and its molecular formula C_22_H_20_N_4_O_3_ was established on the basis of the (+)-HRESIMS *m*/*z* 389.1626 [M + H]^+^, indicating fifteen degrees of unsaturation. The IR spectrum showed absorption bands for amine (3420 cm^−1^), amide carbonyl (1649 cm^−1^) and aromatic (1418 cm^−1^). The ^13^C NMR (Supplementary Materials, Figure S22), DEPTs and HSQC spectra (Table 3, Supplementary Materials, Figure S25) revealed the presence of two amide carbonyls (δ_C_ 169.8 and 167.7), five quaternary sp^2^ (δ_C_ 148.4, 136.0, 131.1, 127.4, 109.5), nine methine sp^2^ (δ_C_ 128.9, 124.1, 122.5, 120.9, 118.5, 118.3, 117.8, 111.3, 109.8), one oxygen bearing quaternary sp^3^ (δ_C_ 85.9), one oxygen bearing methine sp^3^ (δ_C_ 84.0), two methine sp^3^ (δ_C_ 58.6, 55.1) and two methylene sp^3^ (δ_C_ 41.3, 24.7). The ^1^H NMR spectrum (Table 3, Supplementary Materials, Figure S21), exhibited, besides four NH signals at δ_H_ 10.88, brd (*J* = 1.4 Hz), 7.72, brs, 6.68, brs, and 6.05, s, and, in conjunction with the COSY and HSQC spectra (Table 3, Supplementary Materials, Figures S23 and S24), the proton signals of two 1,2-disubstituted benzene rings at δ_H_ 7.60, d (*J* = 7.9 Hz, H-4; δ_C_ 118.5), 7.33, d (*J* = 7.9 Hz, H-7, δ_C_ 111.3), 7.07, ddd (*J* = 7.9, 7.9, 1.1 Hz, H-6, δ_C_ 120.9), 6.99, ddd (*J* = 7.9, 7.9, 0.5 Hz, H-5, δ_C_ 118.3), and at δ_H_ 7.18, d (*J* = 7.4 Hz, H-4′; δ_C_ 122.5), 7.05, ddd (*J* = 7.8, 7.4, 1.3 Hz, H-6′, δ_C_ 128.9), 6.61, ddd (*J* = 7.8, 7.4, 0.5 Hz, H-5’, δ_C_ 117.8) and 6.54, d (*J* = 7.8 Hz, H-7’, δ_C_ 109.8). That one of the 1,2-disubstituted benzene rings was part of the indole moiety was corroborated by the HMBC correlations of H-4 to C-3 (δ_C_ 109.5), C-6 (δ_C_ 120.9) and C-8 (δ_C_ 136.0), of the amine proton at δ_H_ 10.88, brd (*J* = 1.4 Hz, NH-1) to C-2 (δ_C_ 124.1), C-3, C-8, C-9 (δ_C_ 127.4), and of H-2 (δ_H_ 7.25, d, *J* = 2.3 Hz) to C-3 and C-9 (Table 3 and Figure 5). The presence of a 2,5-disubstituted 1,4-diketopiperazine was supported by the HMBC correlations of NH-13’ (δ_H_ 7.72, brs) to the carbonyl at δ_C_ 167.7 (C-12), the methine carbons at δ_C_ 58.6 (C-11′) and δ_C_ 55.1 (C-11) and the methylene carbon at δ_C_ 24.7 (C-10), of NH-13 (δ_H_ 6.05, s) to the methylene carbon at δ_C_ 41.3 (C-10′), of H-11 (δ_H_ 4.46, t, *J* = 5.1 Hz) to C-10 and C-12, of H-11′ (δ_H_ 4.66, dd, *J* = 11.6, 6.7 Hz) to C-10′ and C-12′. Moreover, the COSY correlations of H-11 to H_2_-10 (δ_H_ 3.06, dd, *J* = 15.7, 6.5 Hz and 3.40, m), and of H-11′ to H_2_-10′ (δ_H_ 1.83, dd, *J* = 13.0, 11.6 Hz and 2.43, dd, *J* = 13.0, 6.7 Hz) (Table 3 and Figure 5) confirmed that the substituents on C-11 and C-11′ are methylene groups. The indole ring system was connected to the 1, 4-diketopiperazine moiety through CH_2_-10 since the HMBC spectrum exhibited correlations of H-11 to C-3, and of H-10 to C-9 (Table 3, Figure 5, Supplementary Materials, Figure S25). The second 1,2-disubstituted benzene ring was part of the 2,3-disubstituted 2,3-dihydro-1*H*-indole ring system since the HMBC spectrum showed correlations of NH-1′ (δ_H_ 6.68,d, *J* = 4.1 Hz) to the oxygenated sp^3^ quaternary carbon at δ_C_ 85.9 (C-3′) and to the quaternary aromatic carbon at δ_C_ 131.1 (C-9′), and also of H-2′ (δ_H_ 5.33, d, *J* = 4.1 Hz,) to C-3′. Since the HMBC spectrum showed correlations of H-2′ to C-10′, as well as of H-10′ to C-3′, it was concluded that the 2, 3-disubstituted 2, 3-dihydro-1*H*-indole ring system was linked to the 1, 4-diketopiperazine moiety through CH_2_-10′. As all of the ^1^H and ^13^C data so far mentioned accounted only for C_22_H_20_N_4_O_2_, which is one oxygen atom less than the molecular formula, the epoxide functionality was placed between C-2′ and C-3′.

**Figure 6:**
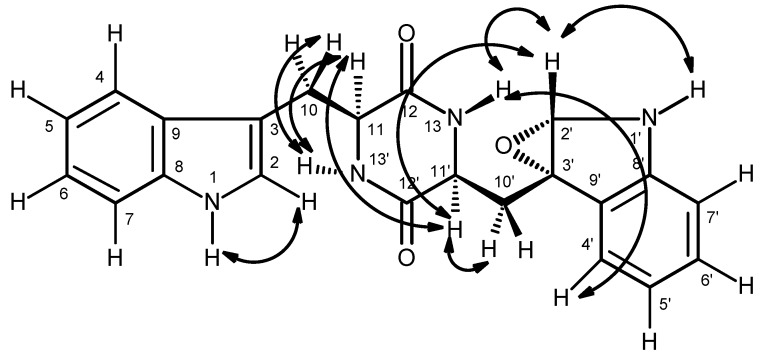
Key NOESY (↔) correlations of compound **8**.

Since compound **8** was obtained as pale yellow viscous mass, its stereochemistry could not be determined by X-ray crystallography. However, as compounds **8** was isolated together with fellutanine A (7) [13,14], it is reasonable to assume that the stereochemistry of C-11 and C-11′ of both compounds are the same. Like compounds **1–3, 5** and **6**, fellutanine A (**7**) and compound **8** must be derived from the same biosynthetic precursor, i.e., l-tryptophan. Consequently, the absolute configurations of C-11 and C-11′ of fellutanine A (**7**) and compound **8** are presumed to be *S*. In an effort to unravel the stereochemistry of C-11, C-11′, and the epoxide bearing carbons (C-2′ and C-3′) of compound **8**, the NOESY experiments and molecular dynamic simulations were carried out. The NOESY spectrum of compound **8** (Table 3 and Figure 6, Supplementary Materials, Figure S26) exhibited correlations of H-11 to H-2, H-4, H-10a, H-11′, NH-13′, therefore confirming the *cis*-relation between H-11 and H-11′. Since the coupling constant between H-11 and H-10a is 6.5 Hz, H-10a must be in an equatorial and H-11 in axial positions in the major conformation. On the other hand, H-11′ exhibited only correlations to H-11 and H-10′b (δ_H_ 2.43, dd, *J* = 13.0, 6.7 Hz), and H-2′, but not with H-10′a (δ_H_ 1.83, dd, *J* = 13.0, 11.6 Hz) and NH-13, while H-2′ gave correlations to only H-11′ and NH-13, but not to H-10′a or 10′b. The values of the coupling constants of H-11′ to H-10′a (*J* = 11.6 Hz) and to H-10′b ((*J* = 6.7 Hz) indicated that H-11′ and H-10′a are “*anti*”, while H-11′ and H-10′b are “gauche”. These data indicated that H-2′ is in the same face as H-11′ and points to the opposite direction from H-10′a/10′b. However, these correlations do not allow us to determine the stereochemistry of the epoxide. Surprisingly, the NOESY spectrum also shows strong correlation of H-4′ to NH-13.

**Figure 7:**
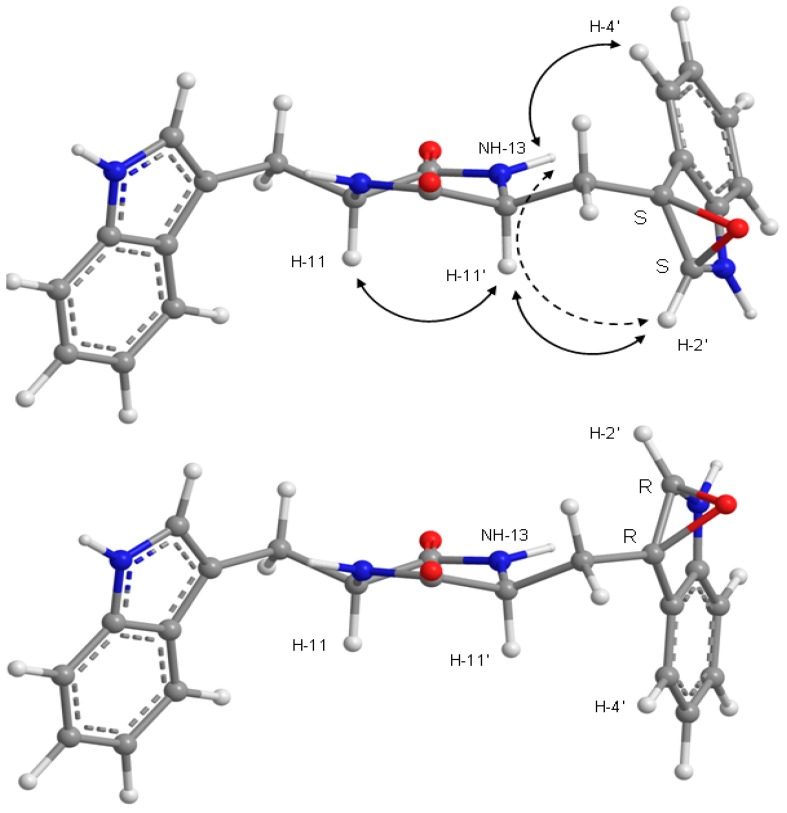
The two possible epoxide configurations for **8** in their lowest RHF/6-21G total energy conformation. Solid lines indicate direct NOESY correlations, explained by the *S*/*S* stereoisomer and not by the *R*/*R*. The discontinuous line shows how spin diffusion gives rise to an H-2′/NH-13 NOESY cross-peak.

Furthermore, a strong NOESY cross-peak between H-11 and H-11′ of compound **8**, and in conjunction with conformational search, molecular dynamics and ab initio molecular modeling, showed that both amide bonds in the diketopiperazine ring are cis and that both amino acids have the same stereochemistry for their α-carbons. This type of six-membered ring is thermodynamically stable because resonance compensates for the extra energy of the amide cis configurations [17], when compared to the more normal trans configuration. Nevertheless, cis peptide bonds occur naturally even in linear biological proteins [18]. NOESY cross-peaks and molecular modeling also aided the assignment of the absolute configurations to the epoxide carbon atoms of **8**. The minimal energy conformations for the *R*/*R* and *S*/*S* models are presented in Figure 7, showing how the epoxide oxygen points outwards in both cases for minimal repulsion. Conformational analysis was based mainly on the combinations of the three staggered conformations for C-10′/C-11′ bond and two for the C-3′/C-10′ bond. These six conformers differ by less than 7 kcal/mol (RHF/6-21G total energy), independently of the configuration of the epoxide. Of all the conformations, the most stable conformation of the 2′*S*/3′*S* epoxide actually explains simultaneously the observed NOESY correlations of H-4′ to NH-13 and of H-2′ to H-11′ (Table 3 and Figure 6, Supplementary Materials, Figure S26). On the other hand, none of the *R*/*R* conformations justifies the NOESY data without assuming unreasonable spin-diffusion. The assignment of the *S*/*S* isomer for the epoxide carbons of **8** has to assume, however, that there is a spin-diffusion during the mixing time; otherwise, given the proposed structure for **8**, it would not be possible to explain also the H-2′/H-13 NOESY cross-peak. The proximities H-4′/NH-13 and H-2′/H-11′ are physically incompatible with direct H-2′/H-13 NOE proximity. The fact that H-2′/H-11′/NH-13 form a coupled dipolar spin system is perhaps an explanation for the very week H-11′/NH-13 NOESY cross-peak (Table 3 and Figure 6, Supplementary Materials, Figure S26), which is expected to be strong unless some polarization transfer is at play between the three spins.

Taking all of the evidence together, the structure of compound **8** was proposed as fellutanine A 2′*S*, 3′*S*-epoxide. To the best of our knowledge, compound **8** is also a new compound.

Compounds **1**–**8** were tested for their antibacterial activity against Gram-positive (*Escherichia coli* ATCC 25922) and Gram-negative (*Staphyllococus aureus* ATCC 25923) bacteria, as well as for their antifungal activity against filamentous (*Aspergillus fumigatus* ATCC 46645), dermatophyte (*Trichophyton rubrum* ATCC FF5) and yeast (*Candida albicans* ATCC 10231), according to the previously described protocols [19,20]; however, none of the tested compounds exhibited either antibacterial (MIC > 256 μg/mL) or antifungal activities (MIC > 512 μg/mL).

## 3. Experimental Section

### 3.1. General Procedure

Melting points were determined on a Bock monoscope and are uncorrected. Optical rotations were measured on an ADP410 Polarimeter (Bellingham + Stanley Ltd., Tunbridge Wells, Kent, UK). Infrared spectra were recorded in a KBr microplate in a FTIR spectrometer Nicolet iS10 from Thermo Scientific (Waltham, MA, USA) with Smart OMNI-Transmission accessory (Software 188 OMNIC 8.3). ^1^H and ^13^C NMR spectra were recorded at ambient temperature on a Bruker AMC instrument (Bruker Biosciences Corporation, Billerica, MA, USA) operating at 300.13 or 500.13 MHz, and 75.4 or 125.8 MHz, respectively. High resolution mass spectra were measured with a Waters Xevo QToF mass spectrometer (Waters Corporations, Milford, MA, USA) coupled to a Waters Aquity UPLC system. A Merck (Darmstadt, Germany) silica gel GF_254_ was used for preparative TLC, and a Merck Si gel 60 (0.2–0.5 mm) was used for column chromatography.

### 3.2. Extraction and Isolation

The strain KUFA 0702 was isolated from the marine sponge *Mycale* sp., which was collected, by scuba diving at a depth of 15–20 m, from the coral reef at Samaesarn Island (12°34′36.64′′ N 100°56′59.69′′ E) in the Gulf of Thailand, Chonburi Province, in February 2015. The sponge was washed with 0.06% sodium hypochlorite solution for 1 min, followed by sterilized seawater 3 times, and then dried on sterile filter paper, cut into small pieces (5 × 5 mm), and placed on a malt extract agar (MEA) medium containing 70% seawater and 300 mg/L of streptomycin sulfate. After incubation at 28 °C for 7 days, the hyphal tips were transferred onto a slant MEA and maintained as pure culture for further identification. The fungus was identified as *Neosartorya glabra* (Fennell & Raper) Kozak based on morphological characteristics such as colony growth rate and growth pattern on standard media, namely Czapek′s agar, Czapek yeast autolysate agar and malt extract agar. Microscopic characteristics including size, shape and ornamentation of ascospores were examined under light and scanning electron microscopes. This identification was supported by sequence analysis of the β-tubulin, calmodulin and actin genes as described in the previous report [21]. *Neosartorya glabra* was also confirmed by sequence analysis of the internal transcribed spacer (ITS) gene, according the procedure previously described by us [11]. Its gene sequences were deposited in GenBank with accession numbers KU955860. The pure cultures were deposited as KUFA 0702 at Kasetsart University Fungal Collection, Department of Plant Pathology, Faculty of Agriculture, Kasetsart University, Bangkok, Thailand. The fungus was cultured for one week at 28 °C in 5 Petri dishes (i.d. 90 mm) containing 15 mL of potato dextrose agar. In order to obtain the mycelial suspension, the mycelial plugs were transferred to two 500 mL Erlenmeyer flasks containing 250 mL of potato dextrose broth, and then incubated on a rotary shaker at 150 rpm at 28 °C for 7 days. Forty 1000-mL Erlenmeyer flasks, each containing 300 g of cooked rice, were autoclaved at 121 °C for 15 min, and then inoculated with 25 mL of mycelial suspension of *N. glabra*, and incubated at 28 °C for 30 days, after which the moldy rice was macerated in ethyl acetate (20 L total) for 7 days, and then filtered with filter paper. The ethyl acetate solution was concentrated under reduced pressure to yield 98.2 g of crude ethyl acetate extract, which was dissolved in 1000 mL of CHCl_3_, and then washed with H_2_O (3 × 500 mL). The organic layers were combined and dried with anhydrous Na_2_SO_4_, filtered and evaporated under reduced pressure to give 71.2 g of the crude chloroform extract, which was applied on a column of silica gel (420 g), and eluted with mixtures of petrol-CHCl_3_ and CHCl_3_–Me_2_CO, 250 mL fractions were collected as follows: Frs 1-80 (petrol–CHCl_3_, 1:1), 81-144 (petrol–CHCl_3_, 3:7), 145-201 (petrol–CHCl_3_, 1:9), 202-356 (CHCl_3_–Me_2_CO, 9:1), 357-398 (CHCl_3_–Me_2_CO, 7:1), and 399-410 (Me_2_CO). Frs 85-105 were combined (2.04 g) and purified by TLC (silica gel G_254_, CHCl_3_–Petrol–EtOAc–HCO_2_H, 8:1:1:0.01) to give 11 mg of ergosta-4,6,8 (14), 22-tetraen-3-one. Fr 207 (1.14 g) was applied over a column chromatography of Sephadex LH-20 (10 g) and eluted with MeOH and a mixture of MeOH: CH_2_Cl_2_ (1:1), wherein 20 mL subfractions were collected as follows: sfrs 1–90 (MeOH), and 91–145 (MeOH: CH_2_Cl_2_, 1:1). Sfrs 53–61 were combined (19.5 mg) and recrystallized in MeOH to give 16.8 mg of ergosterol 5,8-endopeoxide. Sfrs 62-90 were combined (53.2 mg) and purified by TLC (silica gel G_254_, CHCl_3_-Petrol-EtOAc-HCO_2_H, 8:1:1:0.01) to give 11.2 mg of **1**. Frs 206–212 were combined (4.88 g) and applied over a column chromatography of Si gel (45 g) and eluted with mixture of petrol–CHCl_3_, CHCl_3_–Me_2_CO and Me_2_CO, wherein 100 mL subfractions were collected as follows: sfrs 1–51 (petrol–CHCl_3_, 1:1), 52-107 (petrol–CHCl_3_, 3:7), 108–164 (petrol–CHCl_3_, 1:9), 165–190 (CHCl_3_–Me_2_CO, 9.5:0.5), 191–310 (CHCl_3_–Me_2_CO, 9:1). Sfrs 83–164 were combine (53.4 mg) and recrystallized in MeOH to give 27.6 mg of ergosterol 5,8-endopeoxide. Sfr 166 (38.8 mg) was recrystallized in Me_2_CO to give 8.7 mg of **1**. Frs 213–245 were combined (3.61 g) and applied over a column chromatography of Sephadex LH-20 (10 g) and eluted with MeOH, wherein 60 sfrs of 20 mL were collected. Sfrs 31–51 were combined and purified by TLC (silica gel G_254_, CHCl_3_–Me_2_CO–HCO_2_H, 4:1:0.01) to give 9.7 mg of **3** and 13.1 mg of **4**. Frs 246–257 were combined (1.44 g) and recrystallized in MeOH to give 23.7 mg of helvolic acid. Frs 273–287 were combined (621.0 mg) and purified by TLC (silica gel G_254_, CHCl_3_–Me_2_CO–HCO_2_H, 7:3:0.03) to give 12.1 mg of helvolic acid and 32.3 mg of **5**. Frs 363–373 were combined (1.26 g) and applied over a column chromatography of Sephadex LH-20 (10 g) and eluted with MeOH, wherein 60 subfractions of 20 mL were collected. Sfrs 22–54 were combined (91.2 mg) and purified by TLC (silica gel G_254_, CHCl_3_–Me_2_CO–HCO_2_H, 9.5:0.5:0.03) to give 14.7 mg of **6** and 10 mg of **2**. Frs 374–398 were combined (1.37 g) and purified by TLC (silica gel G_254_, CHCl_3_–Me_2_CO–HCO_2_H, 3:2:0.03) to give 32.8 mg of **8**. Frs 403–405 were combined (2.49 g) and applied over a column chromatography of Sephadex LH-20 (10 g) and eluted with MeOH, wherein 112 sfrs of 20 mL were collected. Sfrs 90–112 were combined (24.9 mg) and purified by TLC (silica gel G_254_, CHCl_3_–Me_2_CO–HCO_2_H, 9.5:0.5:0.03) to give 20.7 mg of **7**.

#### 3.2.1. Satoryglabramide A (**5**)

White crystal, mp 146–148 °C (CHCl_3_-Me_2_CO); ${\left[\alpha \right]}_{D}^{20}$ +34.6 (*c* 0.06, Me_2_CO); IR (KBr) ν_max_ 3447, 3060, 3028, 2920, 2850, 1655, 1622, 1587, 1526, 1453, 1415, 1300, 1261, 1173 cm^−1^; ^1^H and ^13^C NMR (see Table 1); HRESIMS *m*/*z* 511.2365 (M + H)^+^ (calculated for C_30_H_31_N_4_O_4_, 511.2345).

#### 3.2.2. Satoryglabramide B (**6**)

White solid, mp 190–192 °C (CHCl_3_–Me_2_CO); ${\left[\alpha \right]}_{D}^{20}$ +42.8 (*c* 0.05, Me_2_CO); IR (KBr) ν_max_ 3417, 3058, 2924, 2852, 1649, 1620, 1588, 1526, 1454, 1418, 1302, 1263, 1101 cm^−1^; ^1^H and ^13^C NMR (see Table 2); HRESIMS *m*/*z* 550.2501 (M + H)^+^ (calculated for C_32_H_32_N_5_O_4_, 550.2454).

#### 3.2.3. Fellutanine A Epoxide (**8**)

Pale yellow viscous mass; ${\left[\alpha \right]}_{D}^{20}$ +13.9 (*c* 0.07, Me_2_CO); IR (KBr) ν_max_ 3420, 2922, 1649, 1416, 1188, 1047, 1025, 996 cm^−1^; ^1^H and ^13^C NMR (see Table 3); HRESIMS *m*/*z* 389.1626 (M + H)^+^ (calculated for C_22_H_21_N_4_O_3_, 389.1614).

### 3.3. X-ray Crystal Structure of Sartoryglabramide A (**5**)

A single crystal of sartoryglabamide A was mounted on a cryoloop using paratone. X-ray diffraction data was collected at room temperature with a Gemini PX Ultra equipped with CuK_α_ radiation (λ = 1.54184 Å). The crystal was orthorhombic, space group P2_1_2_1_2_1_, cell volume 5459.8(2) Å^3^ and unit cell dimensions *a* = 15.1792(3) Å, *b* = 18.7674(5) Å and *c* = 19.1659(3) Å (uncertainties in parentheses). There are two molecules per unit cell with calculated density of 1.242 g/cm^3^. The structure was solved by direct methods using SHELXS-97 and refined with SHELXL-97 [22]. Carbon, nitrogen and oxygen atoms were refined anisotropically. Hydrogen atoms were either placed at their idealized positions using appropriate HFIX instructions in SHELXL and included in subsequent refinement cycles or were directly found from difference Fourier maps and were refined freely with isotropic displacement parameters. The refinement converged to R (all data) = 10.02% and wR2 (all data) = 15.26%. The absolute structure could not be established with confidence (flack *x* parameter 0.3(4)).

Full details of the data collection and refinement and tables of atomic coordinates, bond lengths and angles, and torsion angles have been deposited with the Cambridge Crystallographic Data Centre (CCDC 1483750).

### 3.4. Amino Acids Analysis of Acidic Hydrolysate of Sartoryglabramide A (**5**) and Sartoryglabramide B (**6**)

#### 3.4.1. Acid Hydrolysis

The stereochemistry of the amino acids was determined by analysis of the acidic hydrolysate from **5** and **6**. Compound **5** or **6** (5.0 mg) was dissolved in 6 N HCl (5 mL) and heated at 110 °C, in a furnace, for 24 h in a sealed glass tube. After cooling to room temperature, the solution was dried under N_2_ for 24 h, reconstituted in MeOH for HPLC-MS (200 µL), filtered through a 4 mm PTFE Syringe Filter F2504-4 of 0.2 µm pore size (Thermo Scientific, Mumbai, India), and then analyzed by HPLC equipped with a chiral column.

#### 3.4.2. Chiral HPLC Analysis

The HPLC system consisted of Shimadzu LC-20AD pump, equipped with a Shimadzu DGV-20A5 degasser, a Rheodyne 7725i injector fitted with a 20 µL loop, and a SPD-M20A DAD detector (Kyoto, Japan). Data acquisition was performed using Shimadzu LCMS Lab Solutions software, version 3.50 SP2. The chiral column used in this study was Chirobiotic T (15 cm × 4.6 mm I.D., particle size 5 µm) manufactured by ASTEC (Whippany, NJ, USA). The mobile phase composition was MeOH: H_2_O (80:20 *v*/*v*), all were LC-MS grade solvents obtained from Sigma-Aldrich Co. (St. Louis, MO, USA). The flow rate was 1.0 mL/min and the UV detection wavelength was 210 nm. Analyses were performed at room temperature in an isocratic mode. All standards of pure amino acid enantiomers were purchased from Sigma-Aldrich Co. (St. Louis, MO, USA). The elution order of the enantiomers of all the standards amino acids was confirmed by injecting the solutions of enantiomeric mixtures, and then each enantiomer separately. Working solutions of single enantiomeric amino acids were prepared by dissolution in MeOH at the concentration of 1 mg/mL (10 µL sample injection), while the enantiomeric mixtures were prepared by mixing equal aliquots of each enantiomer (20 µL sample injection). Mix HPLC analyses of the acidic hydrolysate with standard amino acids (co-injection) confirmed the stereochemistry of the amino acids of **5** and **6**.

#### 3.4.3. Molecular Mechanics Conformation Analysis of Fellutanine A Epoxide (**8**)

Molecular simulations for structure **8** were carried out in ChemBio3D Ultra 14 (Perkin-Elmerm, Waltham, MA, USA). Stochastic and dihedral driver conformational search, with MMFF force field energy minimization, was done for both *S*/*S* and *R*/*R* isomers of **8**, followed by ab initio RHF/6-21G energy re-minimization of the lowest energy conformations using CS GAMESS interfaced by ChemBio3D. The PCM solvent model for DMSO was used on the ab initio minimizations.

## 4. Conclusions

Although there are few reports of the constituents of *N. glabra*, this is the first study of the secondary metabolites from the marine-derived strain of this fungus. It is interesting to point out that even though some common fungal metabolites previously isolated from other members of this and related genera, such as ergosta-4,6,8 (14), 22-tetraen-3-one, ergosterol 5,8-endoperoxide, helvolic acid, aszonalenin, takakiamide, (3*R*)-3-(1*H*-indol-3-ylmethyl)-3,4-dihydro-1*H*-1,4-benzodiazepine-2,5-dione (**2**) and fellutanine A (**7**), compound **4** was only described as a synthetic intermediate obtained by cyclocondensation of l-proline with isatoic acid anhydride [12]. Moreover, this is the first report on isolation of the cyclopeptides (sartoryglabramides A and B) from the genus *Neosartorya*. In addition, despite the fact that compounds **1**–**8** did not exhibit antimicrobial activities in our assay protocols, it does not mean that they do not possess any other relevant biological activities. It is also worth mentioning that several cyclopeptides have been shown to possess antifungal and antibacterial activities, however, their potencies depend on the stereochemical configurations of the amino acids constituents [23]. Therefore, it is not surprising that the stereochemistry of the amino acids constituents of both sartoryglabramides A (**5**) and B (**6**) could play an important role in their (lack of) antimicrobial activities. Therefore, it is necessary to further examine the isolated metabolites in other target-based assay protocols.

## Figures and Tables

**Table 1:** ^1^H and ^13^C NMR (DMSO, 500 MHz and 125 MHz) and HMBC assignment for **5**.

	Position	δ_C_, Type	δ_H_, (*J* in Hz)	COSY	HMBC
Anthranilic acid	1	166.5, C	-		
	2	124.8, C	-		
	3	126.6, CH	7.55, dd (7.7, 1.3)	H-4	C-1, 5, 7
	4	122.4, CH	7.16, dd (7.9, 7.7)	H-3, 5	C-2, 6
	5	130.4, CH	7.48, ddd (7.9, 7.9, 1.4)	H-4, 6	C-3, 7
	6	120.4, CH	8.31, dd (7.9, 0.5)	H-5	C-2, 4
	7	136.5, C	-		
	NH-8	-	9.40, s	-	C-2, 6, 9
Phe-I	9	168.8, CO	-		
	10	55.2, CH	4.36, ddd (8.4, 7.8, 5.3)	H-11, NH-21	C-9, 11, 12
	11a	34.7, CH_2_	2.97, dd (13.9, 8.4)	H-10, 11b	C-9, 10, 12, 13, 17
	b		3.23, dd (13.9, 5.3)	H-10, 11a	C-9, 10, 12, 13, 17
	12	138.3, C	-		
	13	129.6, CH	7.08, dd (7.4, 1.4)	H-14	C-11, 15, 17
	14	128.0, CH	7.19, dd (7.4, 7.4)	H-14, 15	C-12, 16
	15	126.0, CH	7.18, dd (7.4, 7.4)	H-14, 16	C-13, 17
	16	128.0, CH	7.19, dd (7.4, 7.4)	H-15, 17	C-12, 14
	17	129.6, CH	7.08, dd (7.4, 1.4)	H-16	C-11, 13, 15
	NH-18	-	8.49, d (7.8)	H-20	C-10, 19
Phe-II	19	169.9, CO	-		
	20	54.4, CH	4.58, ddd (9.8, 8.9, 7.3)	H-21a, b	C-19, 21, 22
	21a	37.1, CH_2_	2.71, dd (13.5, 8.9)	H-21b, 20	C-19, 20, 22, 23, 27
	b		2.94, dd (13.5, 7.3)	H-21a, 20	C-19, 20, 22, 23, 27
	22	137.3, C	-		
	23	129.1, CH	7.14, dd (7.4, 1.4)	H-24	C-25, 27
	24	128.1, CH	7.27, dd (7.4, 7.4)	H-23, 25	C-22, 26
	25	126.3, CH	7.23, dd (7.4, 7.4)	H-24, 26	C-23, 27
	26	128.1, CH	7.27, dd (7.4, 7.4)	H-25, 27	C-22, 24
	27	129.1, CH	7.14, dd (7.4, 1.4)	H-26	C-23, 25
	NH-28	-	7.41, d (9.8)	H-20	C-19, 20, 29
Pro	29	170.2, CO	-		
	30	62.2, CH	4.20, dd (9.8, 2.3)	H-31a, b	C-29, 31, 32
	31a	28.3, CH_2_	1.54, m	H-30, 31b	**-**
	b		2.12, m	H-30, 31a	C-29, 30
	32	24.6, CH_2_	1.89, m	H-31a, b, 32a, b	
	33a	49.4, CH_2_	3.70, dd (17.6, 9.6)	H-32, 33b	C-30, 32
	b		3.63, m	H-32, 33a	
	N-34	-	-		

**Table 2:** ^1^H and ^13^C NMR (DMSO, 500 MHz and 125 MHz) and HMBC assignment for **6**.

	Position	δ_C_, Type	δ_H_, (*J* in Hz)	COSY	HMBC
Anthranilic acid	1	166.4, CO	-		
	2	125.2, C	-		
	3	126.5, CH	7.53, d (7.6)	H-4	C-1, 5, 7
	4	122.6, CH	7.16, dd (7.6, 7.6)	H-3, 5	C-2, 6
	5	130.4, CH	7.48, ddd (8.3, 7.6)	H-4, 6	C-3, 7
	6	120.7, CH	8.27, d (8.3)	H-5	C-2, 4
	7	136.3, C	-		
	NH-8	-	9.25, s		C-2, 6, 9
Trp	9	169.0, CO	-		
	10	54.3, CH	4.52, ddd (7.9, 6.7, 5.9)	H-11, NH-21	C-9, 11, 12, 22
	11a	24.9, CH_2_	3.32, dd (14.7, 5.9)	H-10, 11b	C-9, 10, 12, 13, 20
	b		3.14, dd (14.7, 6.7)	H-10, 11a	C-9, 10, 12, 13, 20
	12	110.2, C	-		
	13	127.7, C	-		
	14	118.5, CH	7.58, d (7.9)	H-15	C-16, 18
	15	118.2, CH	6.98, dd (7.9, 7.5)	H-14, 16	C-13, 17
	16	120.8, CH	7.06, dd (8.0, 7.5)	H-15, 17	C-14, 18
	17	111.3, CH	7.34, d (8.0)	H-16	C-13, 15
	18	136.0, C	-		
	NH-19	-	10.82, brs	H-20	C-12, 13, 18, 20
	20	124.0, CH	7.04, d (1.8)	NH-19	C-13
	NH-21	-	8.42, d (7.9)	H-10	C-9, 22
Phe	22	170.1, CO	-		
	23	54.6, CH	4.61, ddd (10.0, 10.0, 6.4)	H-24a, b	C-24, 32
	24a	37.0, CH_2_	2.66, dd (13.6, 10.0)	H-23, 24b	C-22, 23, 25, 26, 30
	b		2.92, dd (13.6, 6.4)	H-23, 24a	C-22, 23, 25, 26, 30
	25	134.4, C	-		
	26	129.0, CH	7.10, dd (7.7, 1.0)	H-27	C-25
	27	128.1, CH	7.20, m	H-26, 28	C-25
	28	126.3, CH	7.18, m	H-27, 29	
	29	128.1, CH	7.20, m	H-28, 30	C-28
	30	129.0, CH	7.10, dd (7.7, 1.0)	H-29	C-25
	NH-31	-	7.38, d (10.0)	H-23	C-32
Pro	32	170.2, CO	-		
	33	62.1, CH	4.15, dd (9.0, 1.2)	H-34a, b	C-32
	34a	28.3, CH_2_	1.45, m	H-33, 34b	
	b		2.09, m	H-33, 34a	
	35	24.6, CH_2_	1.86, m	H-34a,b, 36a, b	
	36a	49.4, CH_2_	3.55, m	H-35, 36b	
	b		3.67, m	H-35, 36a	
	N-37	-	-		

**Table 3:** ^1^H and ^13^C NMR (DMSO, 300 and 75 MHz), HMBC assignment and NOESY for **8**.

Position	δ_C_, type	δ_H_, (*J* in Hz)	COSY	HMBC	NOESY
2	124.1, CH	7.25, d (2.3)	NH-1	C-3, 9	H-10a, 11 (str), NH-13′
3	109.5, C	-			
4	118.5, CH	7.60, d (7.9)	H-5	C-3, 6, 8	H-10a, 11 (str)
5	118.3, CH	6.99, ddd (7.9, 7.9, 0.5)	H-4, 6	C-7, 9	
6	120.9, CH	7.07, ddd (7.9, 7.9, 1.1)	H-5, 7	C-4, 8	
7	111.3, CH	7.33, d (7.9)	H-6	C-5, 9	
8	136.0, C	-			
9	127.4, C	-			
10a	24.7, CH_2_	3.06, dd (15.7, 6.5)	H-10b, 11	C-3, 9, 11, 12	H-4, 10b, 11, NH-13′
b		3.40, m	H-10a, 11	C-3, 9, 11, 12	H-10a
11	55.1, CH	4.46, t (5.1)	H-10a, 10b	C-3, 10, 12	H-2, 4, 10a, 11′, NH-13′
12	167.7, CO	-			
2′	84.0, CH	5.33, d (4.1)	NH-1′	C-3′, 10′	H-11, NH-13, NH-1′ (str)
3′	85.9, C	-			
4′	122.5, CH	7.18, d (7.4)	H-5′	C-6′, 8′	NH-13
5′	117.8, CH	6.61, ddd (7.8, 7.4, 0.5)	H-4′, 6′	C-7′, 9′	
6′	128.9, CH	7.05, ddd (7.8, 7.8, 1.3)	H-5′, 7′	C-4′, 8′	
7′	109.8, CH	6.54, d (7.8)	H-6′	C-5′, 9′	
8′	148.4, C	-			
9′	131.1, C	-			
10′a	41.3, CH_2_	1.83, dd (13.0, 11.6)	H-10′b, 11	C-11′, 12′	H-10′b
b		2.43, dd (13.6, 6.7)	H-10′a, 11	C-3′	H-10′a, 11′
11′	58.6, CH	4.66, dd (11.6, 6.7)	H-10′a, 10b	C-10′, 12′	H-11, 2′, 10′b
12′	169.8, CO	-			
NH-1	-	10.88, brd (1.4)	H-2	C-2, 3, 8, 9	H-2, 4
NH-1′	-	6.68, d (4.1)	H-2′	C-3′, 9′	
NH-13	-	6.05, s	-	C-10′	H-2′, 4′
NH-13′	-	7.72, brs	-	C-10, 11, 11′, 12	H-10a (str), 11 (str), H-2

## Supplementary Materials

The supplementary materials are available online at www.mdpi.com/1660-3397/14/7/136/s1.
