# Supplementary material for: New Cyclotetrapeptides and a New Diketopiperzine Derivative from the Marine Sponge-Associated Fungus Neosartorya glabra KUFA 0702
Source: Mar Drugs. 2016 Jul 20;14(7):136. doi: 10.3390/md14070136 (PMC4962026; doi:10.3390/md14070136)
Supplement: Supplementary file 1 [file marinedrugs-14-00136-s001.pdf]

Supplementary Materials: New Cyclotetrapeptides and a New Diketopiperzine Derivative from the Marine Sponge-Associated Fungus *Neosartorya glabra* KUFA 0702

War War May Zin, Suradet Buttachon, Tida Dethoup, Carla Fernandes, Sara Cravo, Madalena M. M. Pinto, Luís Gales, José A. Pereira, Artur M. S. Silva, Nazim Sekeroglu and Anake Kijjoa

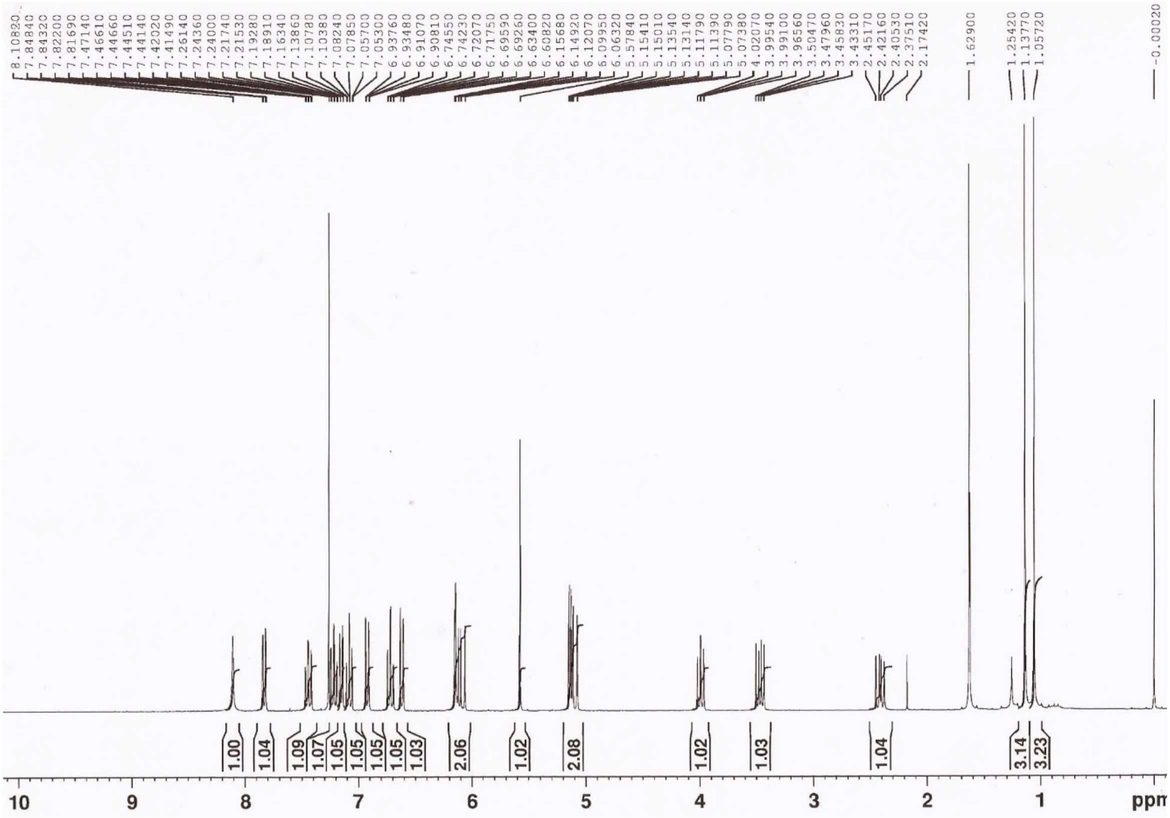

Figure S1. ^1H NMR spectrum of compound **1** ($CDCl_3$, 300.13 MHz).

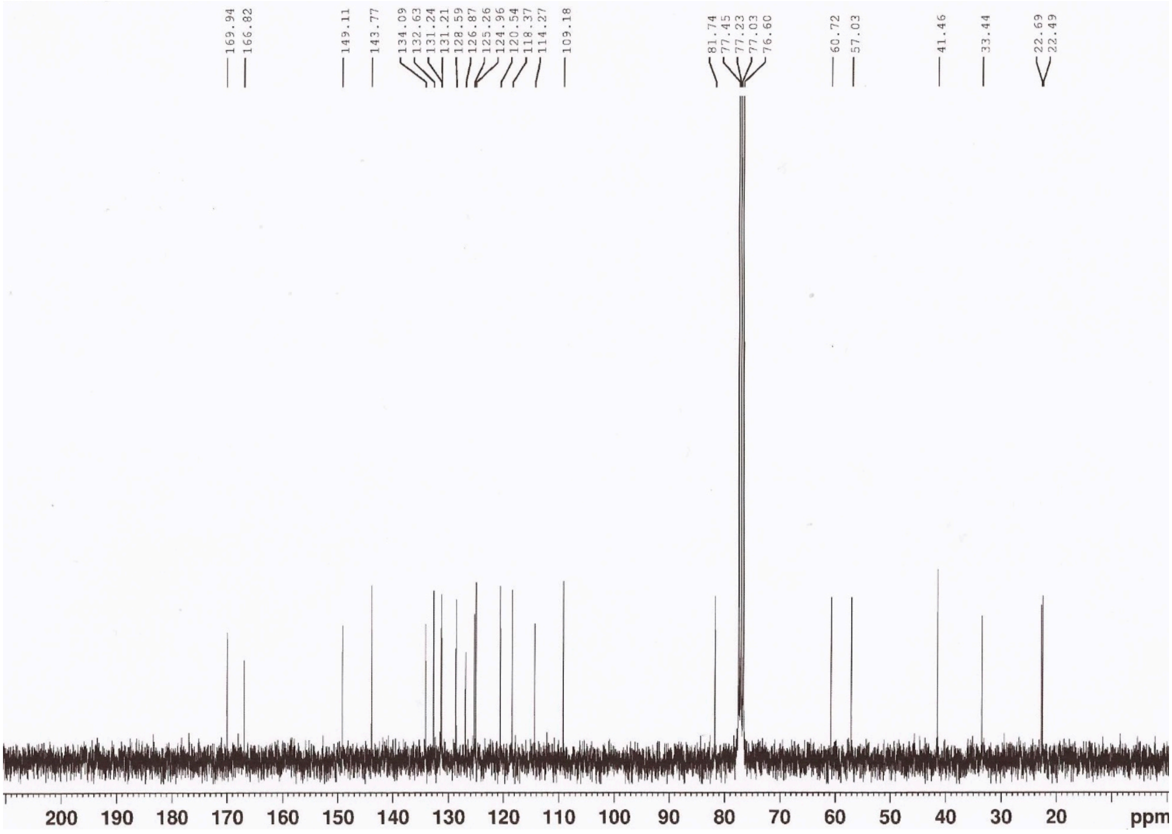

Figure S2. ^{13}C NMR spectrum of compound **1** ($CDCl_3$, 75.4 MHz).

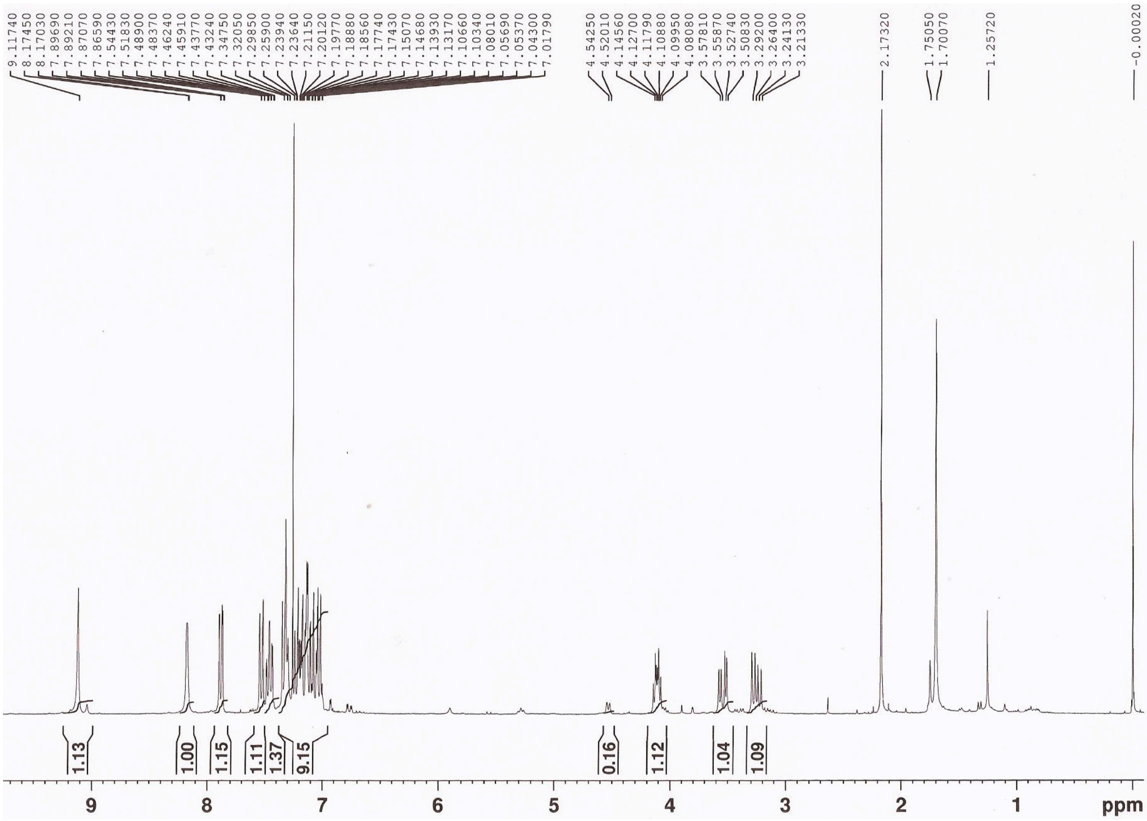

Figure S3. ^{1}H NMR spectrum of compound **2** ($CDCl_3$, 300.13 MHz).

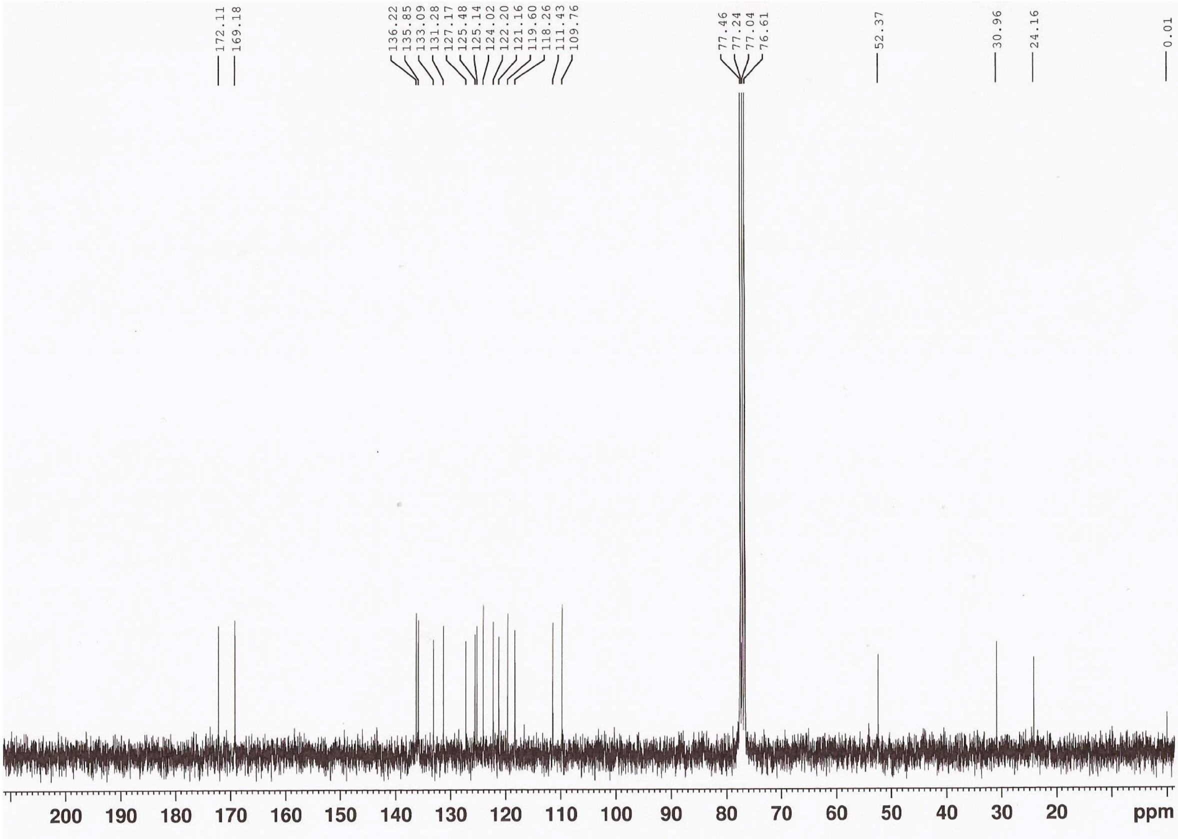

Figure S4. ^{13}C NMR spectrum of compound **2** ($CDCl_3$, 75.4 MHz).

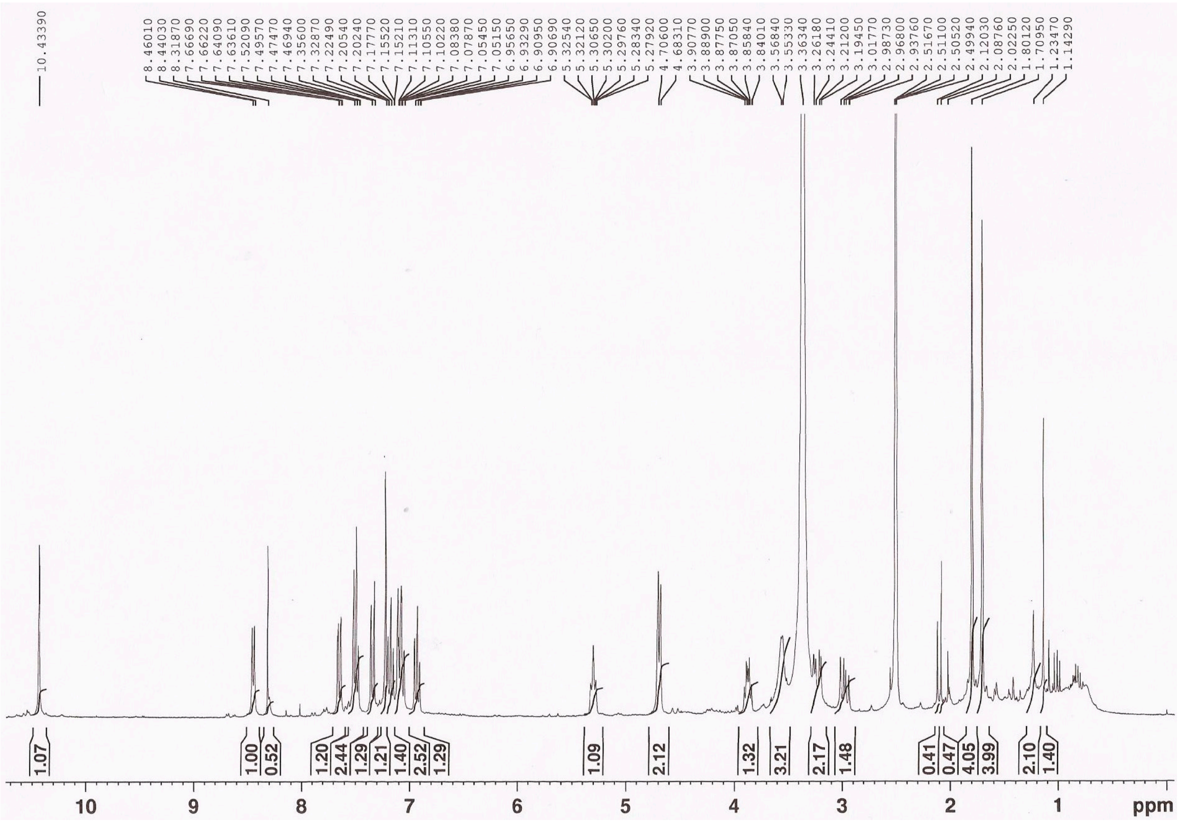

Figure S5. ^{1}H NMR spectrum of compound **3** ($CDCl_3$, 300.13 MHz).

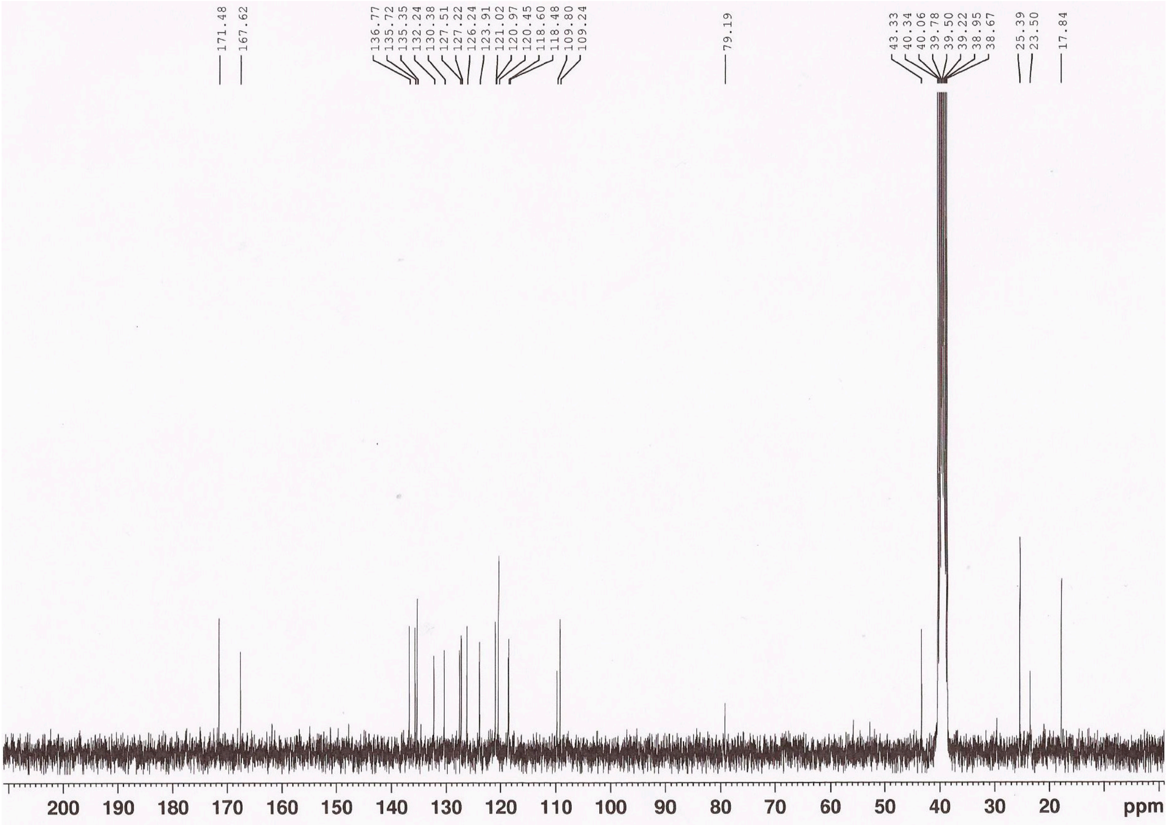

Figure S6. ^{13}C NMR spectrum of compound **3** ($CDCl_3$, 75.4 MHz).

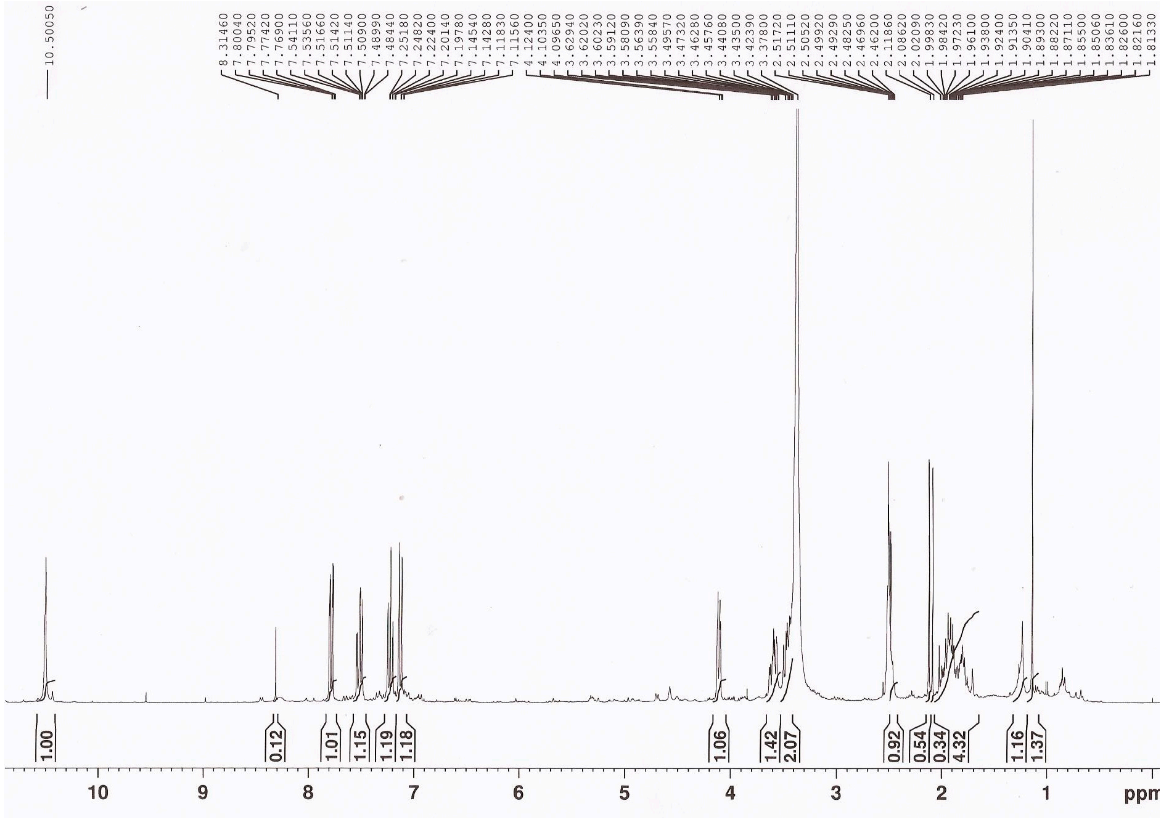

Figure S7. ^{1}H NMR spectrum of compound **4** (DMSO, 300.13 MHz).

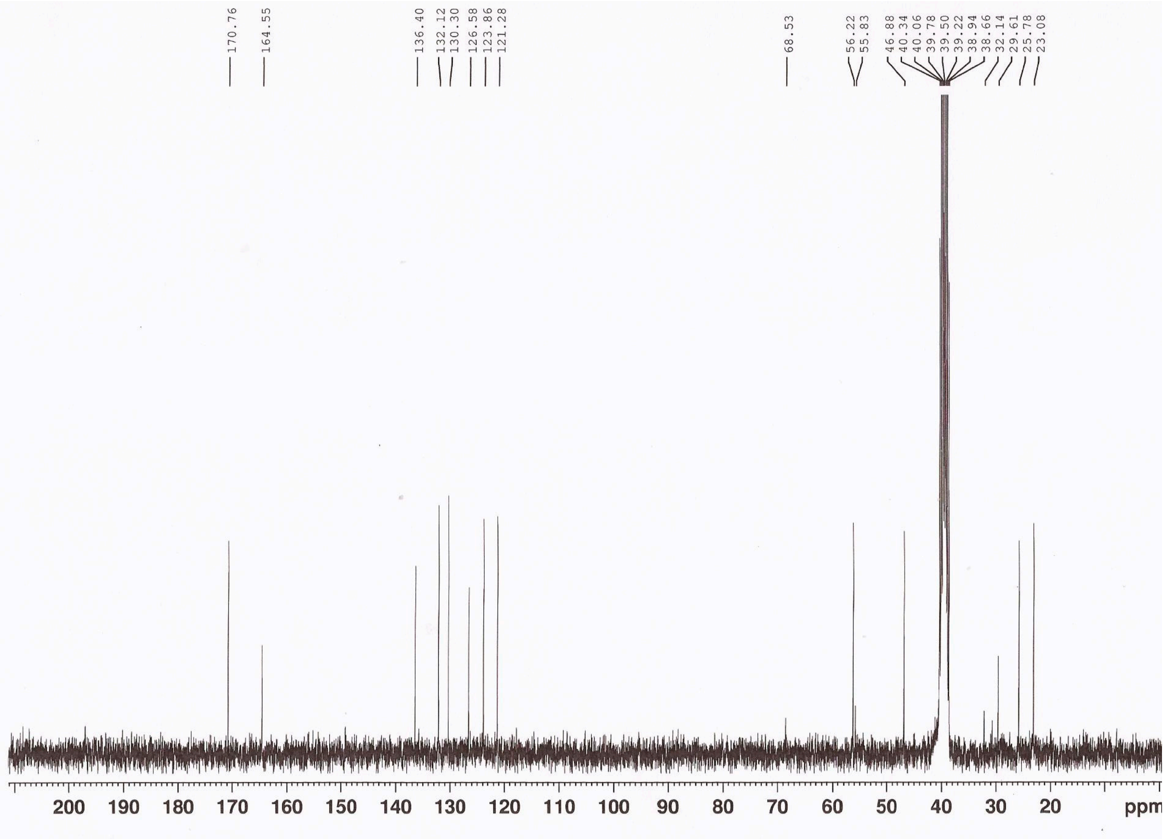

Figure S8. ^{13}C NMR spectrum of compound **4** (DMSO, 75.4 MHz).

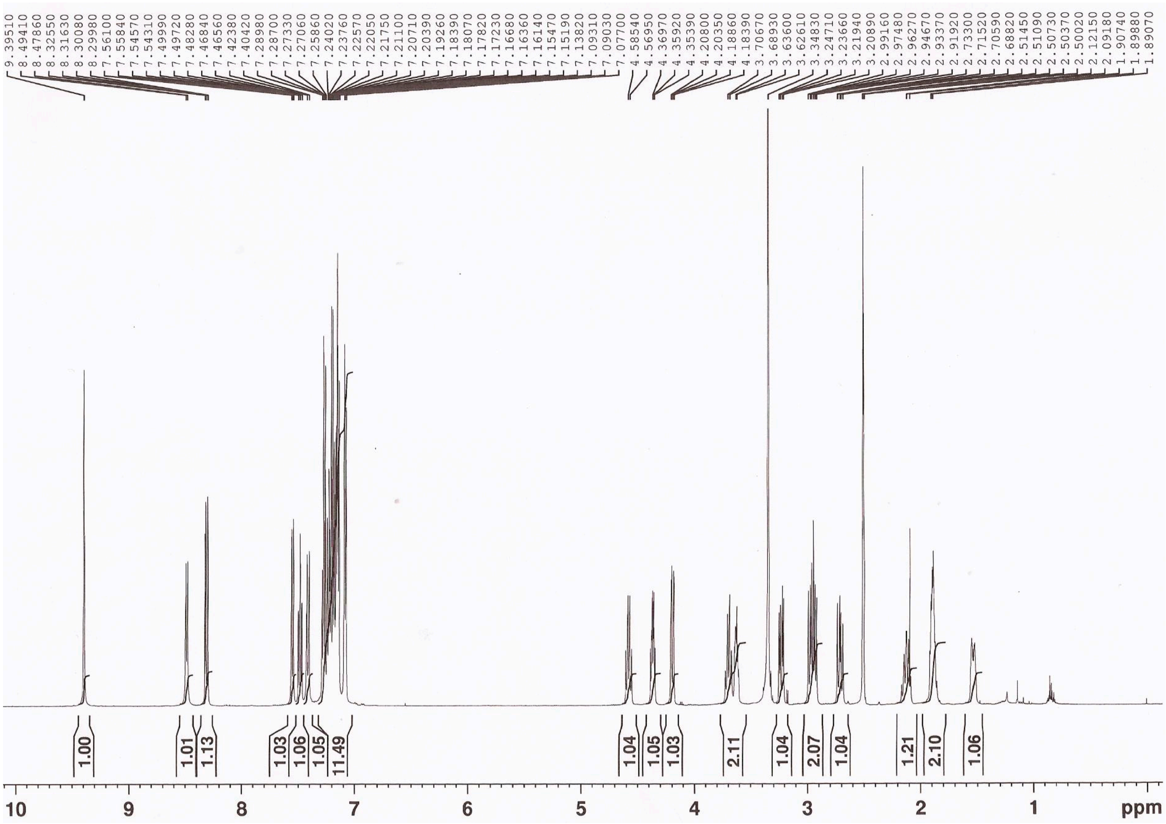

Figure S9. ^{1}H NMR spectrum of compound **5** (DMSO, 500.13 MHz).

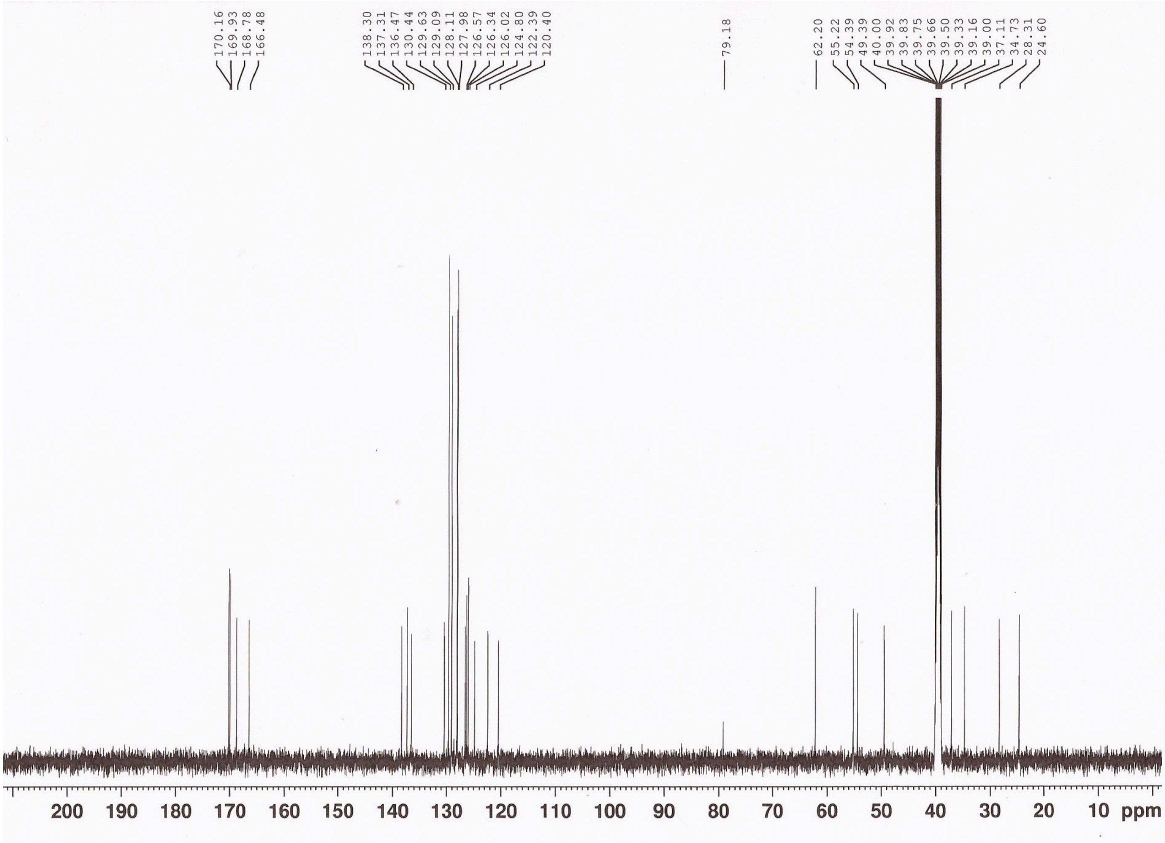

Figure S10. ^{13}C NMR spectrum of compound **5** (DMSO, 125.8 MHz).

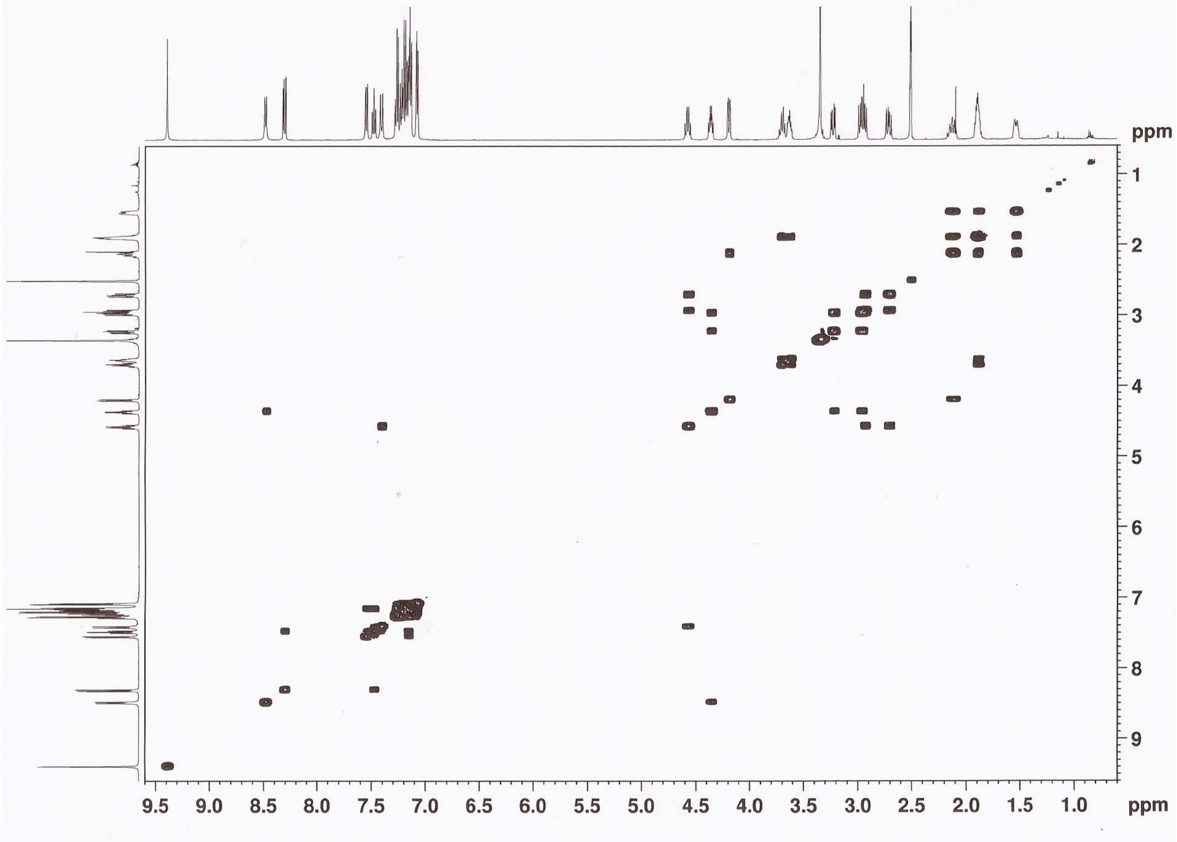

Figure S11. COSY spectrum of compound **5** (DMSO, 500.13 MHz).

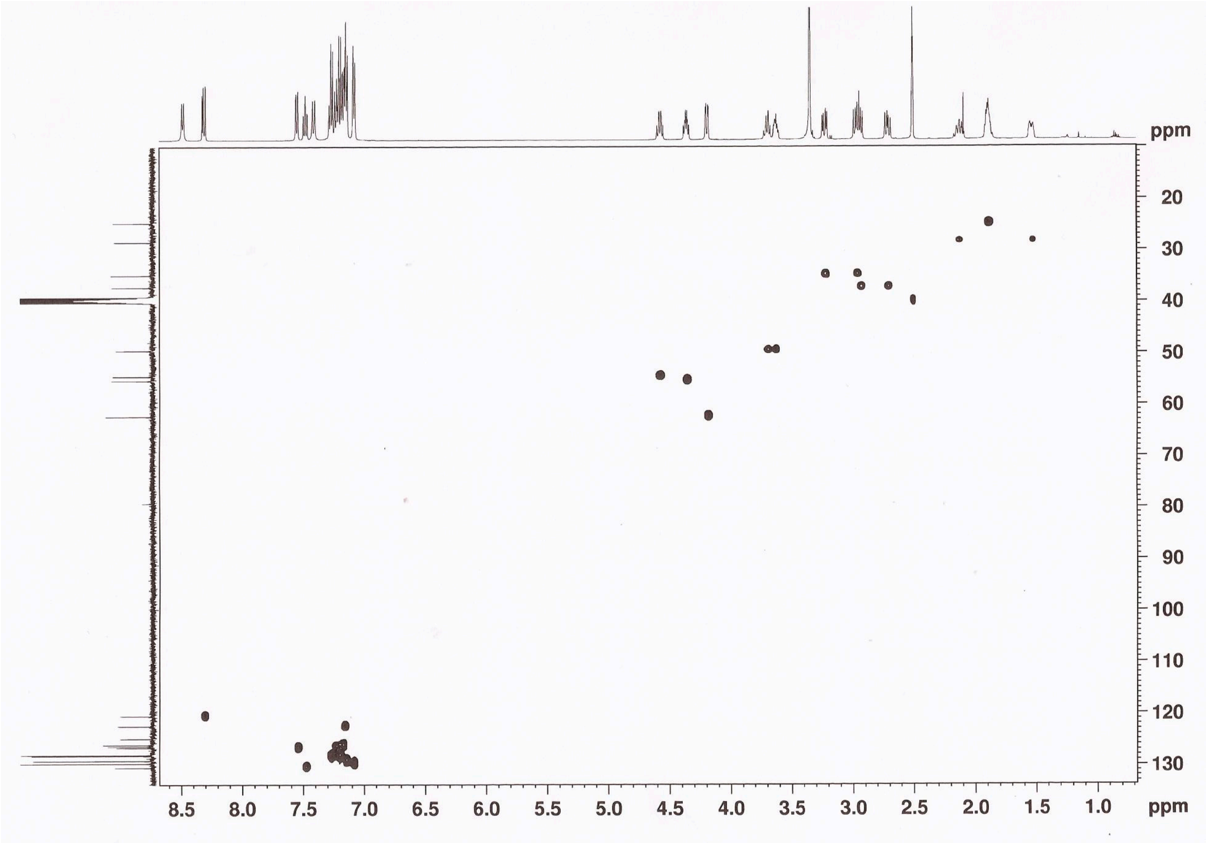

Figure S12. HSQC spectrum of compound **5** (DMSO, 500.13 MHz).

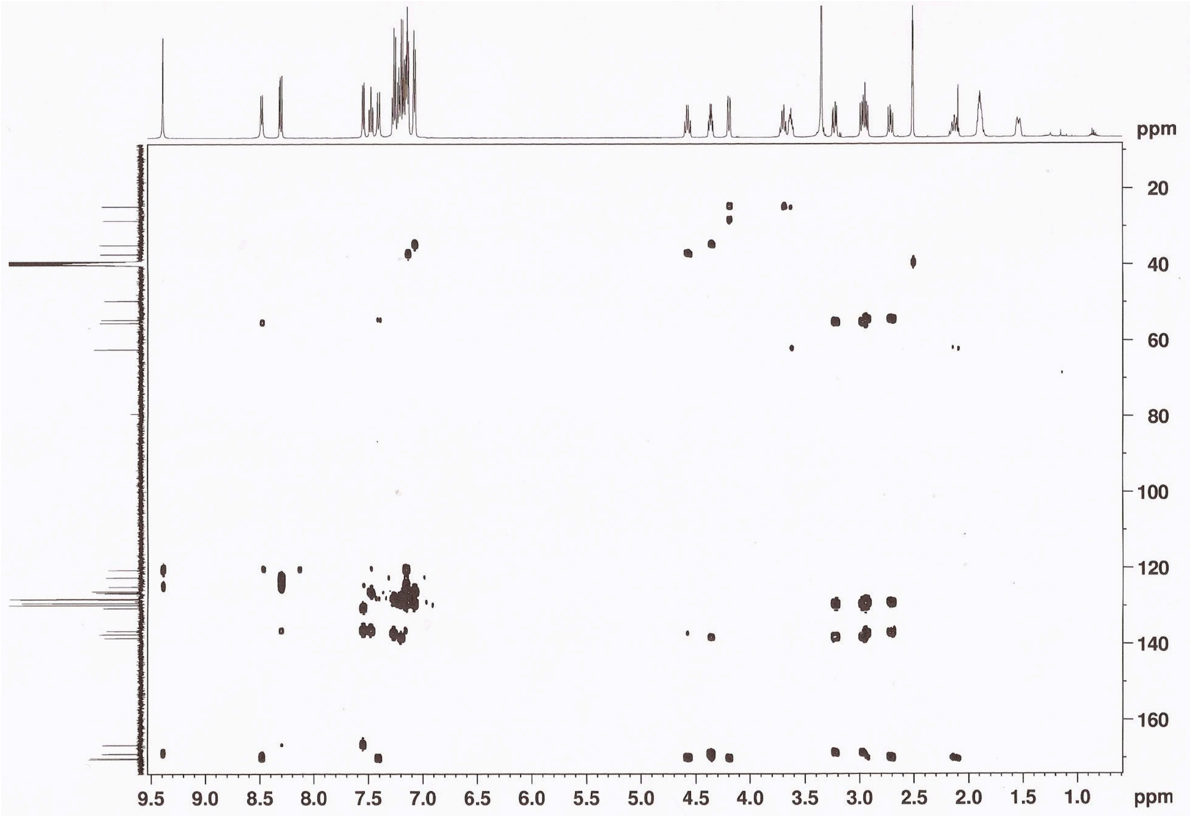

Figure S13. HMBC spectrum of compound **5** (DMSO, 500.13 MHz).

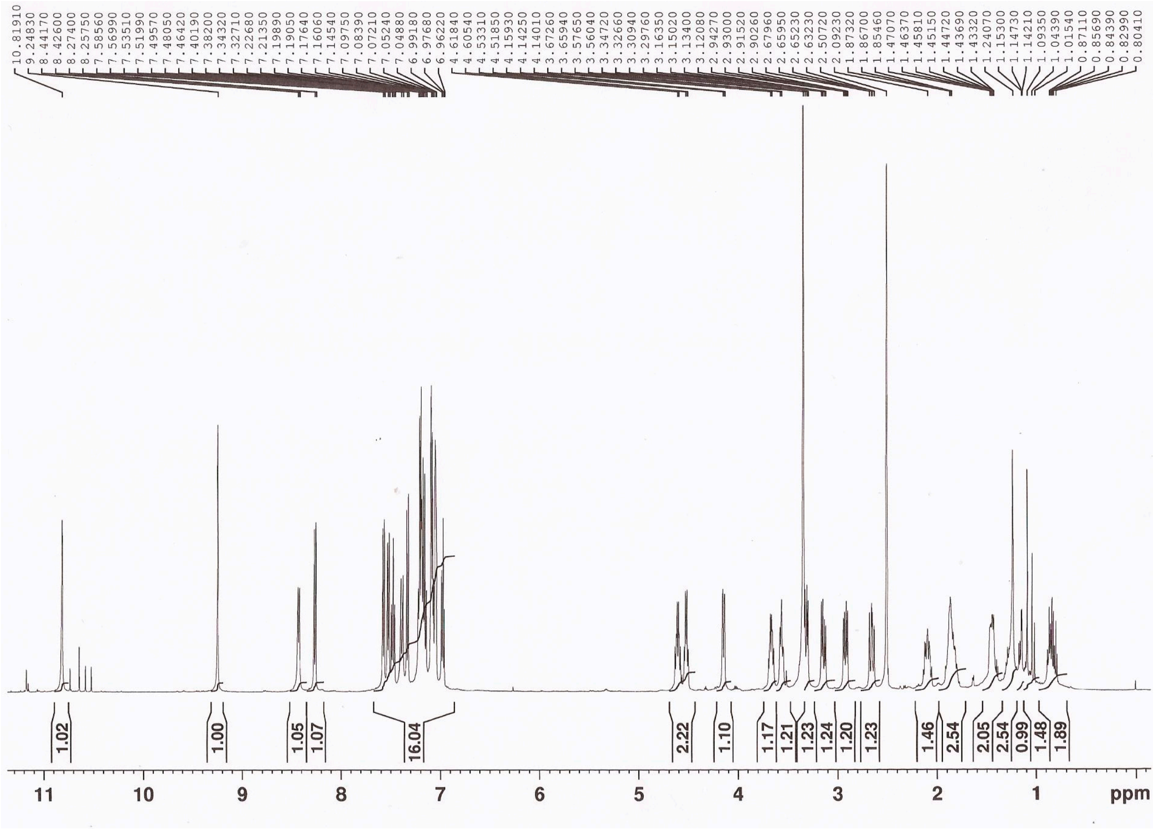

Figure S14. ^{1}H NMR spectrum of compound **6** (DMSO, 500 MHz).

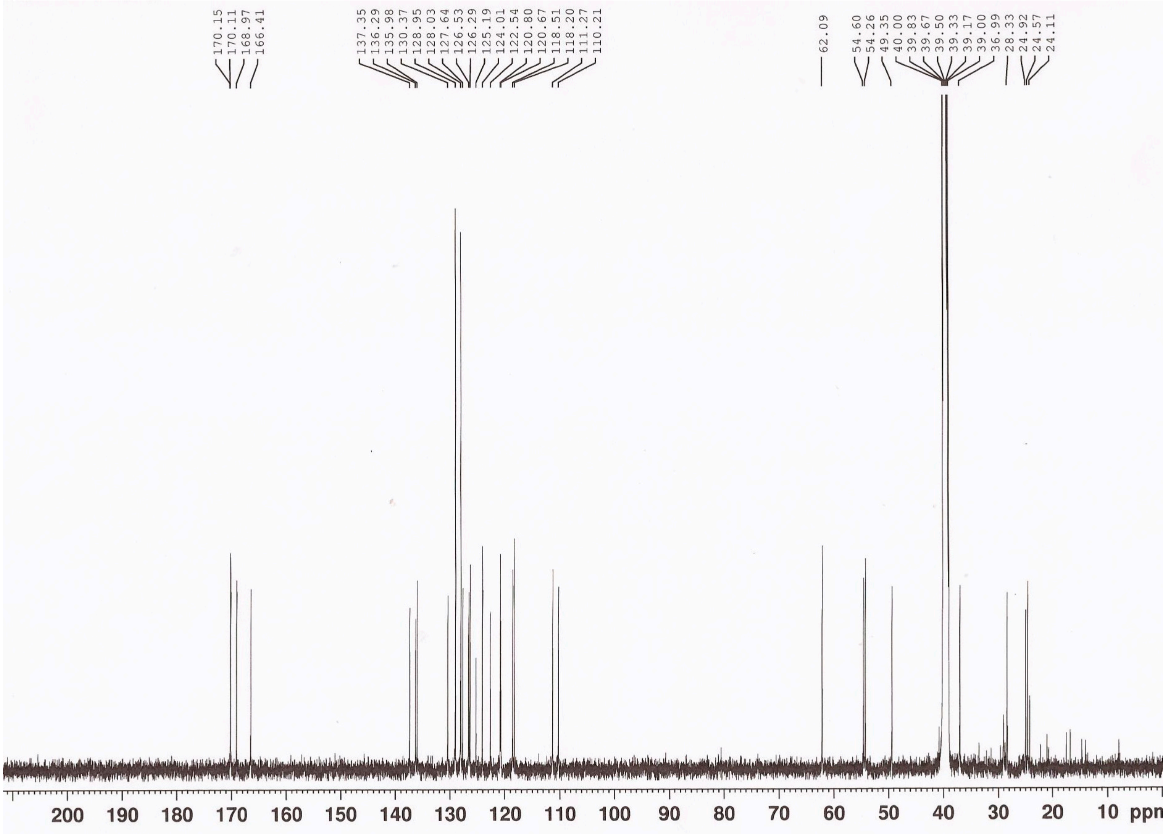

Figure S15. ^{13}C NMR spectrum of compound **6** (DMSO, 125.8 MHz).

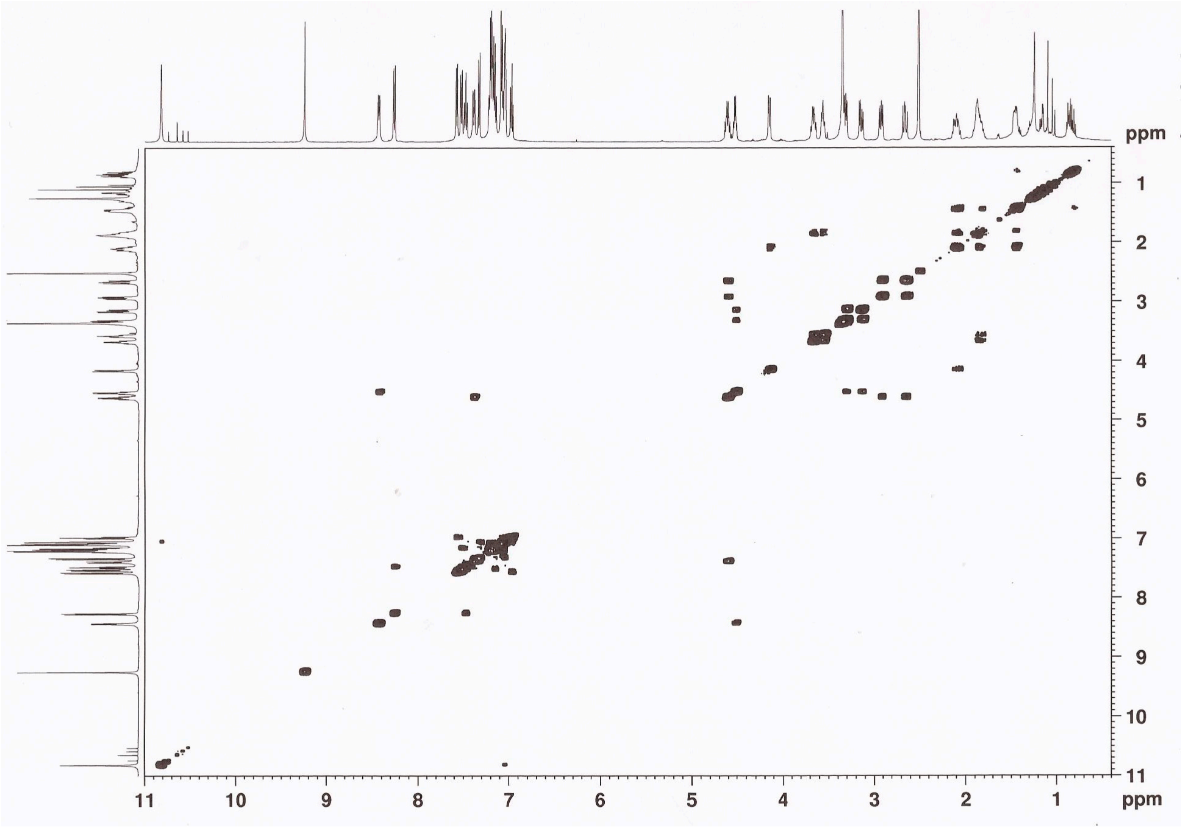

Figure S16. COSY spectrum of compound **6** (DMSO, 500.13 MHz).

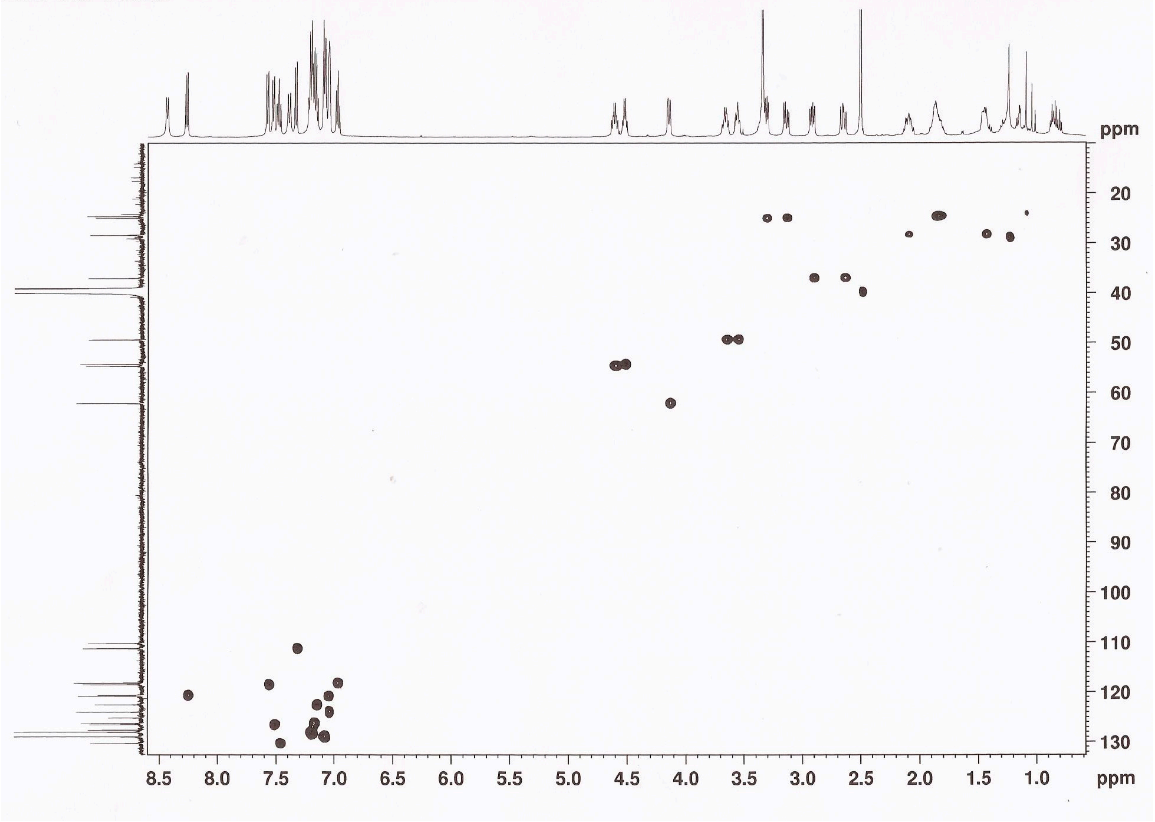

Figure S17. HSQC spectrum of compound **6** (DMSO, 500.13 MHz).

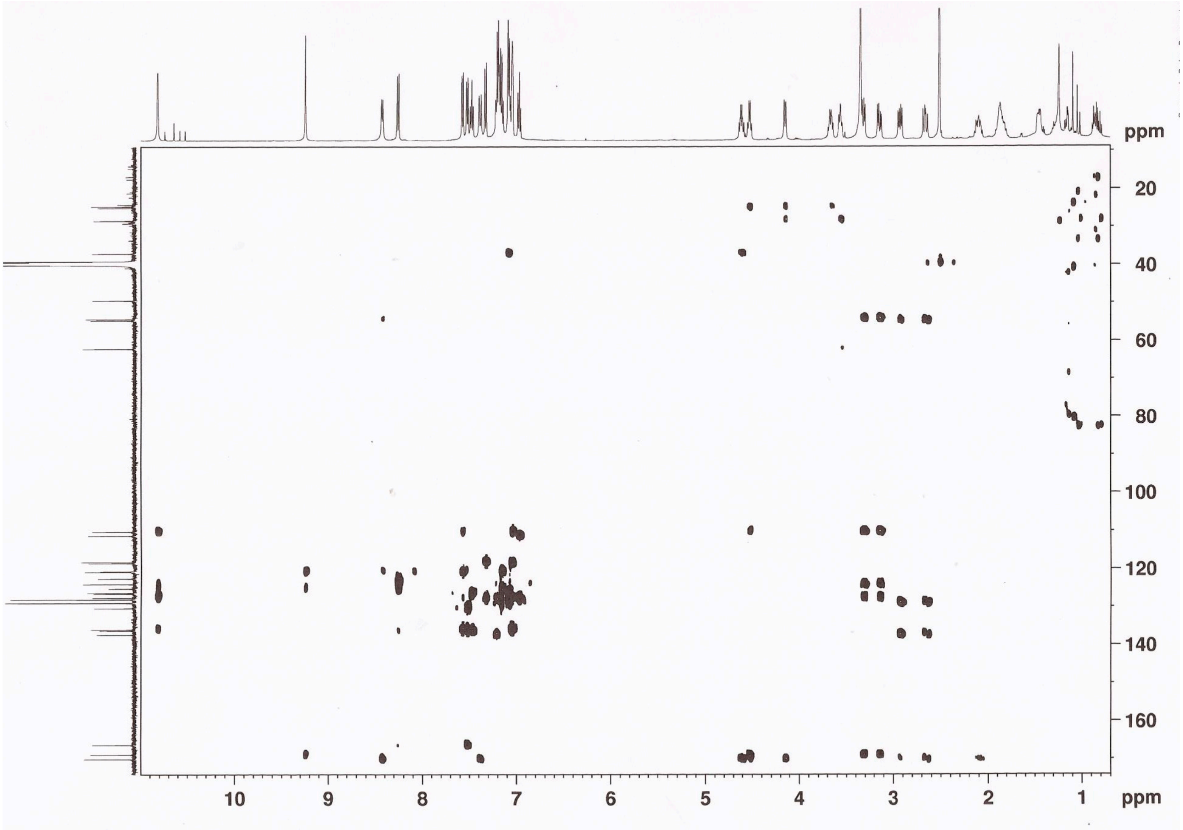

Figure S18. HMBC spectrum of compound **6** (DMSO, 500.13 MHz).

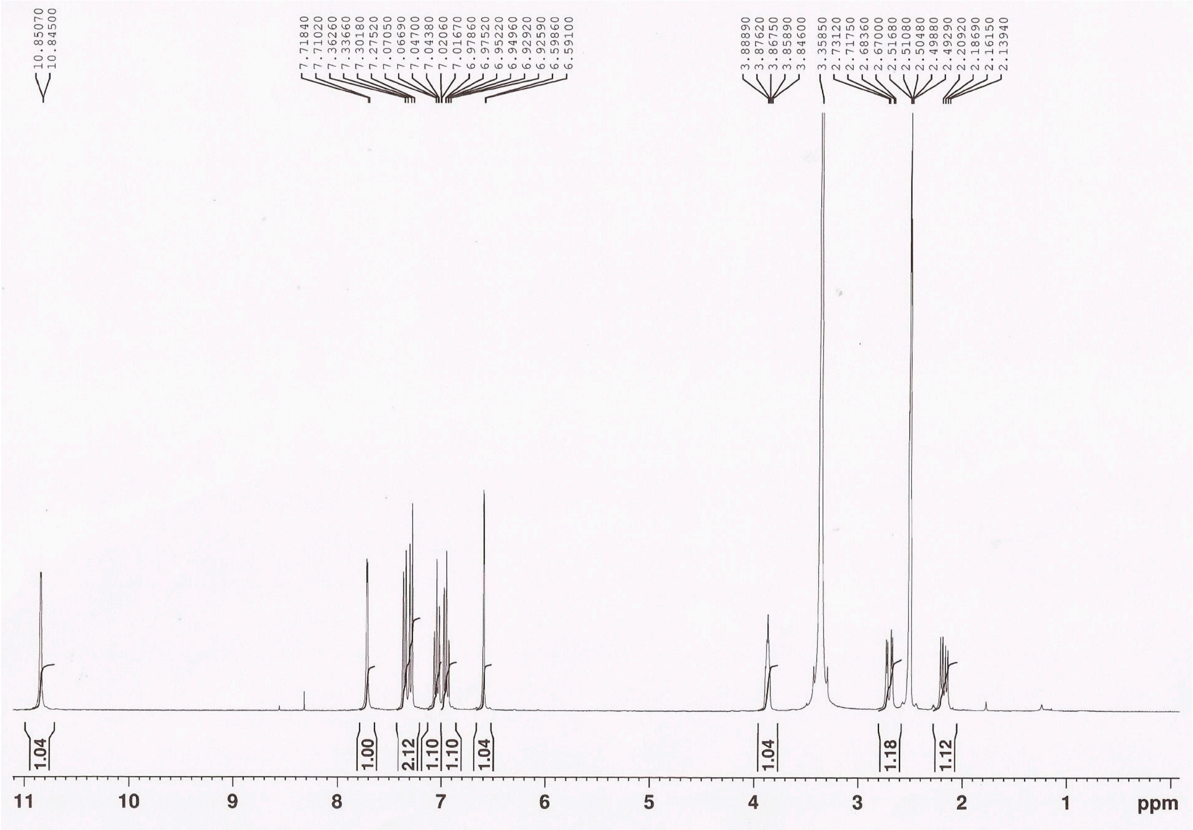

Figure S19. ^{1}H NMR spectrum of compound **7** (DMSO, 300.13 MHz).

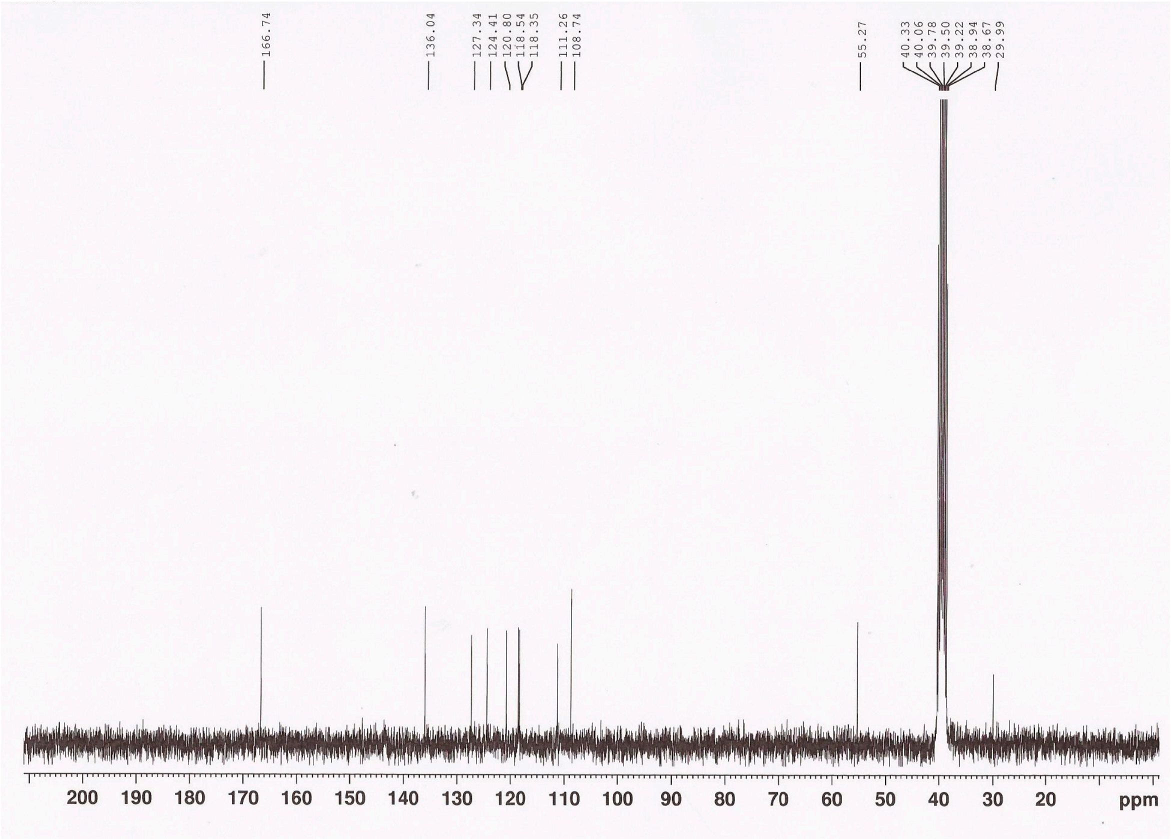

Figure S20. ^{13}C NMR spectrum of compound **7** (DMSO, 75.4 MHz).

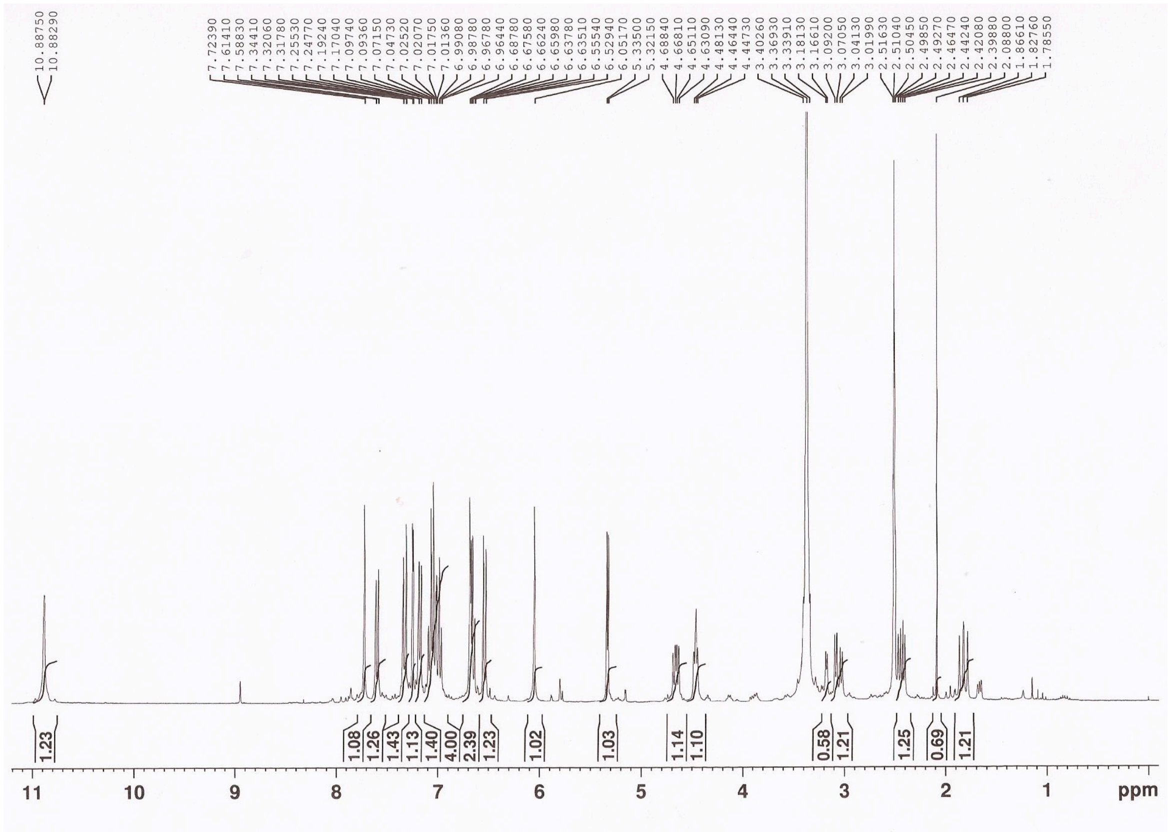

Figure S21. ^{1}H NMR spectrum of compound **8** (DMSO, 300.13 MHz).

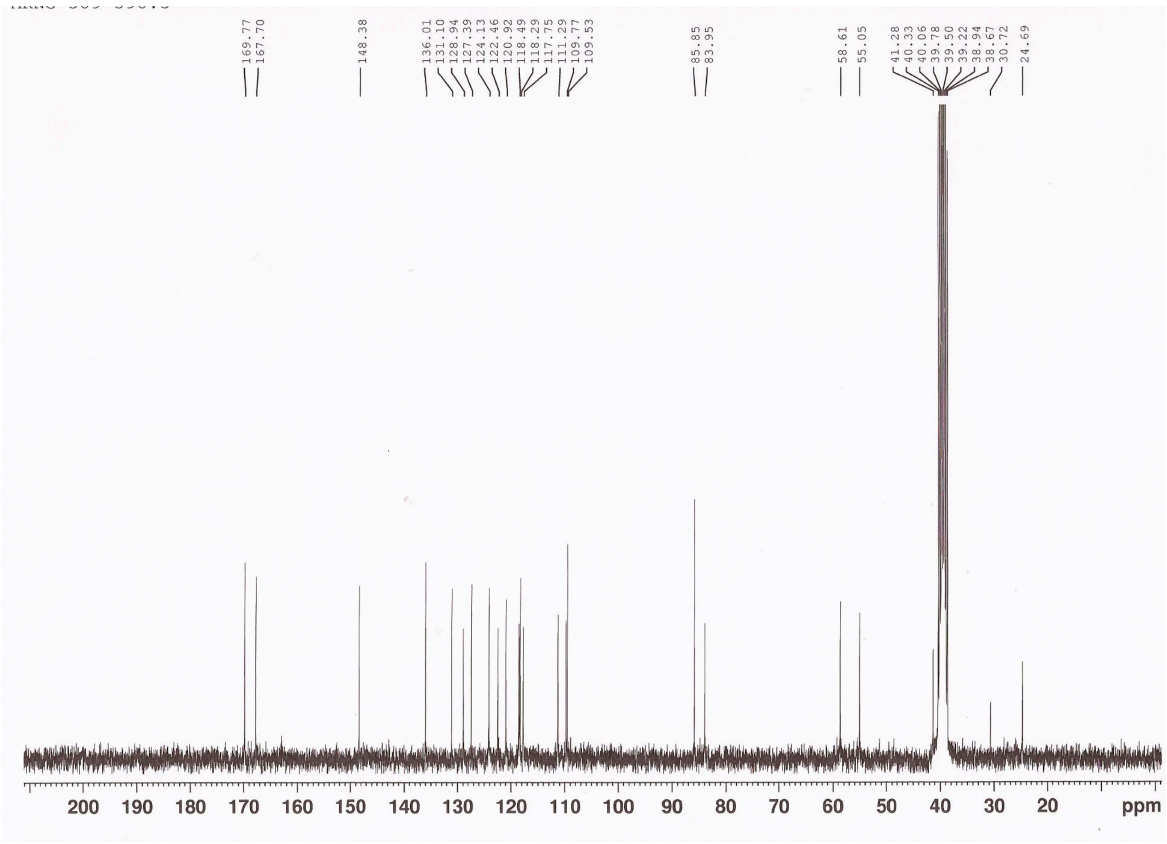

Figure S22. ^{13}C NMR spectrum of compound **8** (DMSO, 75.4 MHz).

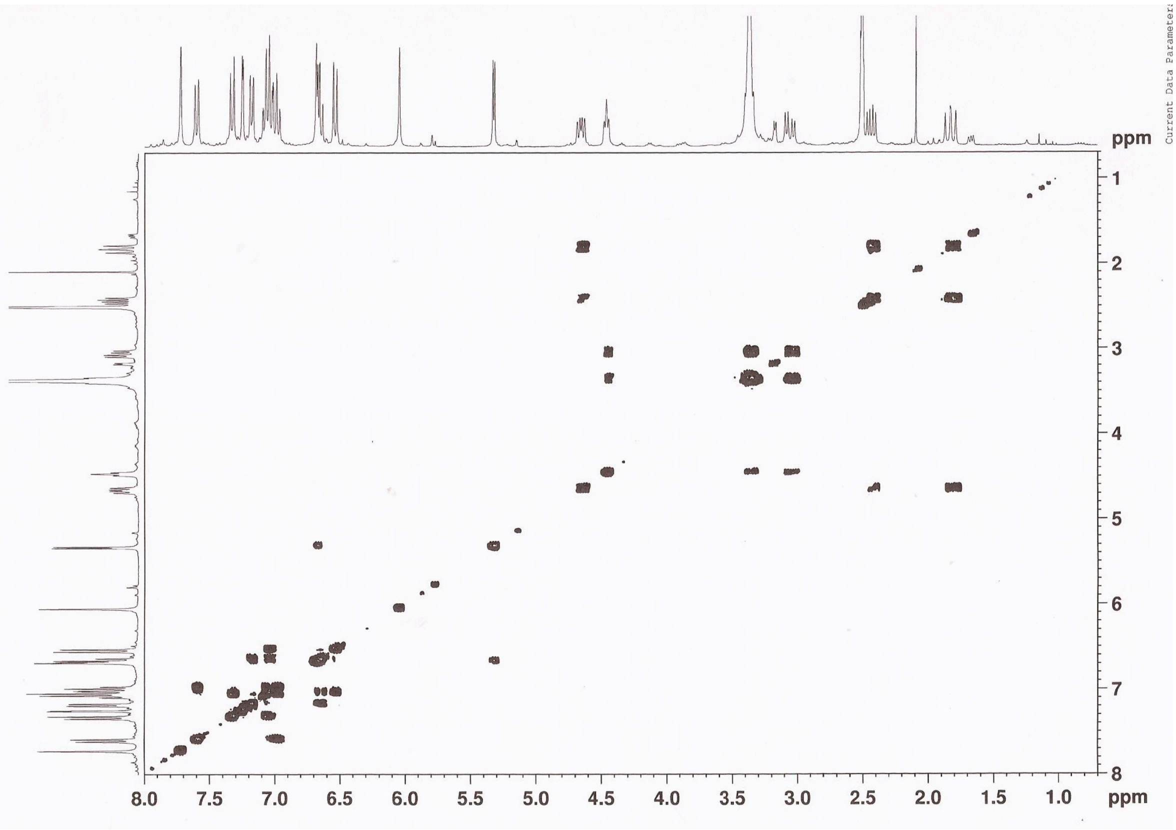

Figure S23. COSY spectrum of compound **8** (DMSO, 300.13 MHz).

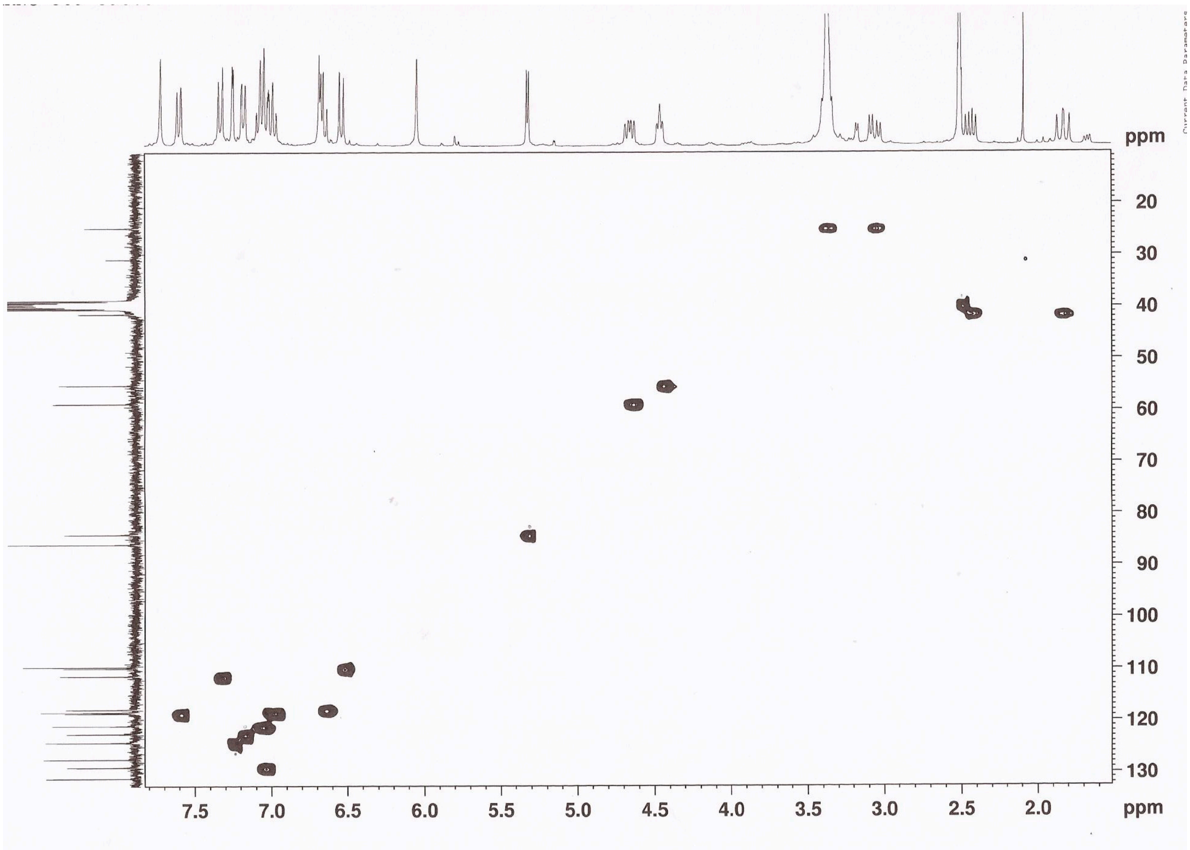

Figure S24. HSQC spectrum of compound **8** (DMSO, 300.13 MHz).

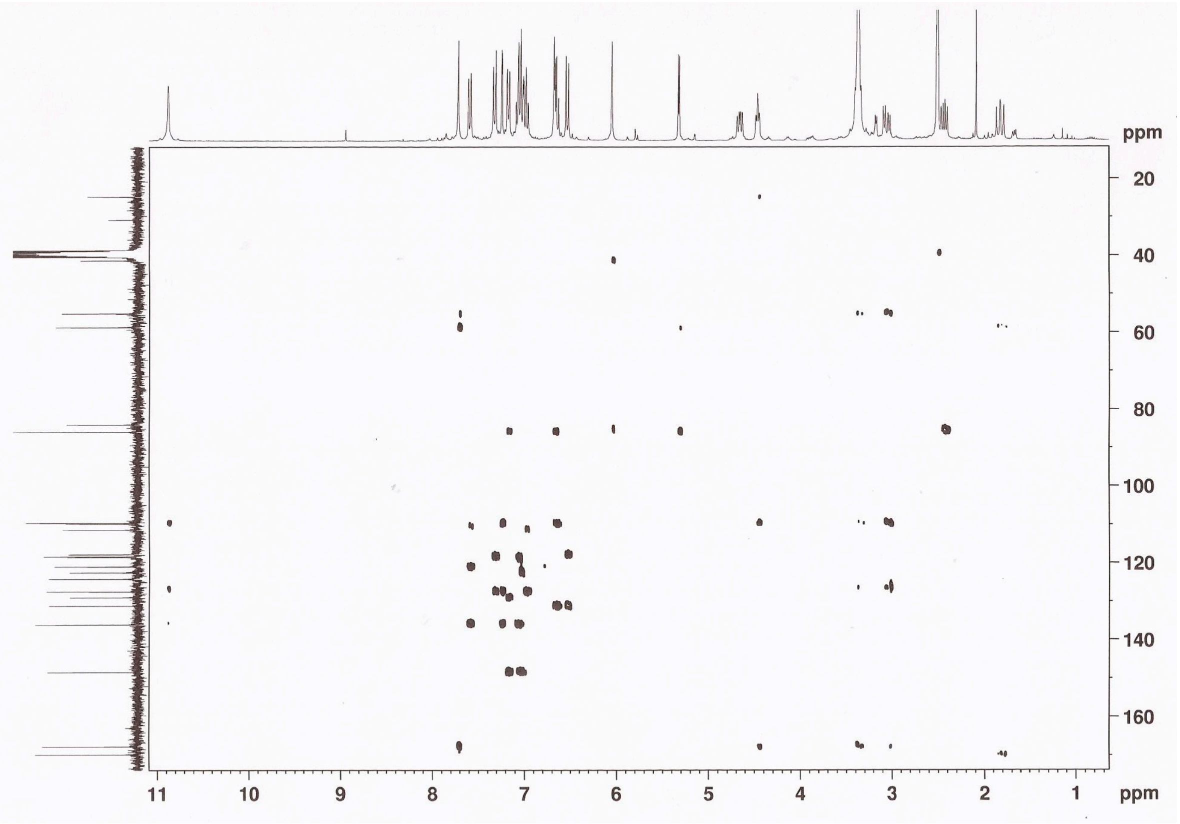

Figure S25. HMBC spectrum of compound **8** (DMSO, 300.13 MHz).

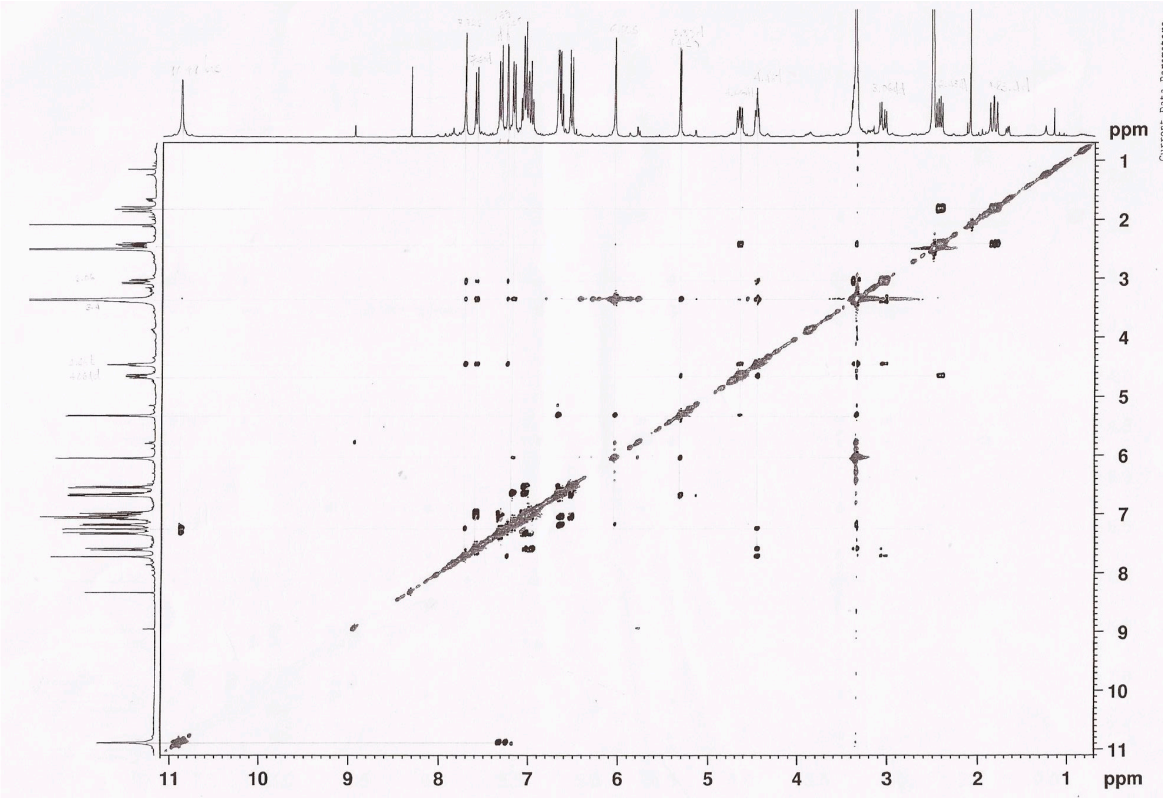

Figure S26. NOESY spectrum of compound **8** (DMSO, 300.13 MHz).

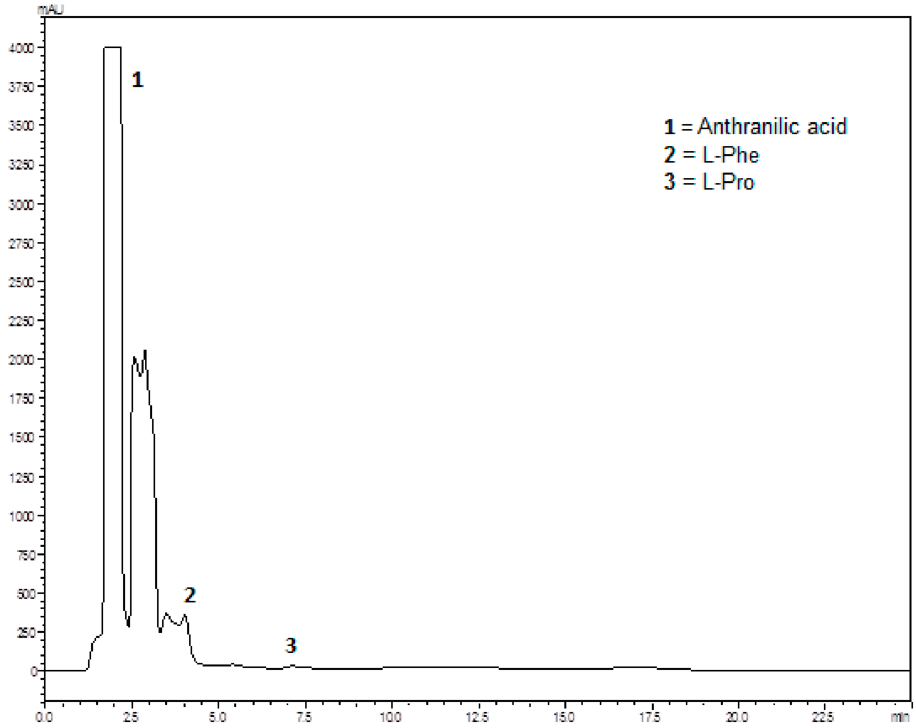

Figure S27. Chromatogram of the acidic hydrolysate of compound **5**. Chromatographic conditions: column, Chirobiotic T; mobile phase, MeOH: H_2O (80:20 *v*/*v*); flow rate, 1.0 mL/min; detection, 210 nm.

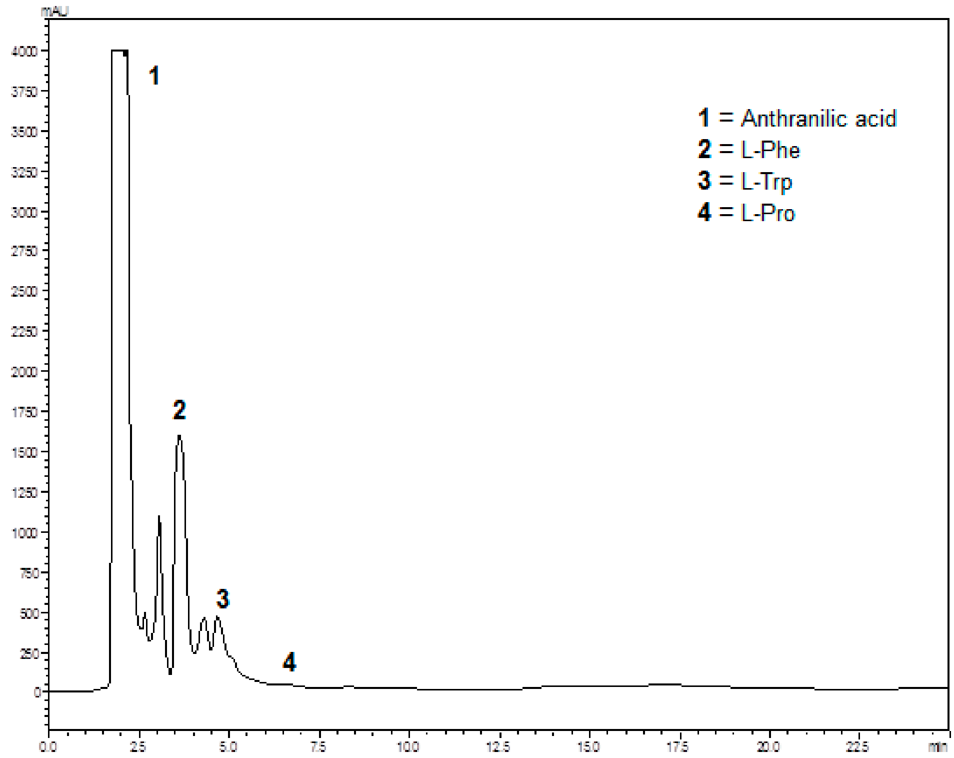

Figure S28. Chromatogram of the acidic hydrolysate of compound **6**. Chromatographic conditions: column, Chirobiotic T; mobile phase, MeOH: H_2O (80:20 *v*/*v*); flow rate, 1.0 mL/min; detection, 210 nm.

Table S1. Chiral HPLC analysis of the acidic hydrolysate of compound **5** and **6**.

	**Retain Time (min)**
L-Phe	3.81
D-Phe	5.00
l-Pro	6.72
D-Pro	20.10
L-Trp	4.51
D-Trp	5.20
Anthranilic acid	1.92
Acidic hydrolysate of **5**	1.91, 2.55, 2.86, 3.49, 3.89, 6.79
Acidic hydrolysate of **5** + DL-Phe (coinjection)	1.87, 2.50, 2.89, 3.68, 5.01, 6.82
Acidic hydrolysate of **5** + DL-Pro (coinjection)	1.96, 2.60, 2.96, 3.52, 3.92, 6.70, 21.09
Acidic hydrolysate of **6**	1.93, 3.07, 3,80, 4.29, 4.60, 6.62
Acidic hydrolysate of **6** + DL-Phe (coinjection)	1.90, 3.10, 3,78, 4.39, 5.04, 6.70
Acidic hydrolysate of **6** + DL-Pro (coinjection)	2.04, 3.02, 3.72, 4.30, 4.60, 6.66, 19.40
Acidic hydrolysate of **6** + DL-Trp (coinjection)	1.93, 2.99, 3.70, 4.29, 4.60, 5.07, 6.33

Chromatographic conditions: column, Chirobiotic T; mobile phase, MeOH: H_2O (80:20 *v*/*v*); flow rate, 1.0 mL/min; detection, 210 nm.
